# 3D-Bioprinted Marine Bacteria for the Degradation of Polyhydroxybutyrate Bioplastics

**DOI:** 10.1021/acsapm.5c03370

**Published:** 2026-04-25

**Authors:** Luying He, Hongyi Cai, Ram S. Gona, Tiana C. Rohe, Manasi Subhash Gangan, Timothy Lai, Diana R. Sullivan, Meredith N. Silberstein, Anne S. Meyer

**Affiliations:** † Department of Biology, 6927 University of Rochester, Rochester, New York 14627, United States; ‡ Materials Science and Engineering, 5922 Cornell University, Ithaca, New York 14853, United States; § Program of Materials Science, University of Rochester, Rochester, New York 14627, United States; ∥ Department of Biochemistry and Biophysics, University of Rochester, Rochester, New York 14627, United States; ⊥ Department of Chemical Engineering, University of Rochester, Rochester, New York 14627, United States; # Sibley School of Mechanical and Aerospace Engineering, Cornell University, Ithaca, New York 14853, United States; ∇ Engineered Living Materials Institute, Cornell University, Ithaca, New York 14853, United States

**Keywords:** biodegradable plastics, 3D bioprinting, engineered living materials, bioplastic-degrading bacteria, marine debris

## Abstract

The severe, long-lasting harm caused by plastic pollution to marine ecosystems and coastal economies has led to the development of biodegradable plastics; however, their limited decomposition in marine environments remains a challenge. Here, technologies are presented for creating 3D-bioprinted living materials as a proof of concept for bioplastic degradation, with specific use in marine environments. The approach developed here integrates the halotolerant bioplastic-degrading bacterium *Bacillus* sp. NRRL B-14911 into alginate-based bio-ink to print an engineered living material (ELM) termed a “bio-sticker.” Quantification of bacteria viability reveals that bioprinted marine bacteria survive within biostickers for more than 3 weeks. The rate at which the biostickers degrade the bioplastic polyhydroxybutyrate (PHB) can be tuned by altering biosticker biomass concentration, bioplastic concentration, or incubation temperature. Biostickers that are transferred to a different PHB sample still retain high biodegradation activity, demonstrating their reusability. Strain sweep oscillatory tests demonstrate that the biostickers display predominantly viscoelastic behavior. Monotonic tensile tests indicate that the elastic modulus and the adhesion of the biostickers are not negatively impacted by bacteria growth or incubation temperature. This work paves the way for the development of ELMs to facilitate the inclusion of bioplastics within the blue economy, promoting the emergence of more sustainable and eco-friendly materials.

## Graphical abstract

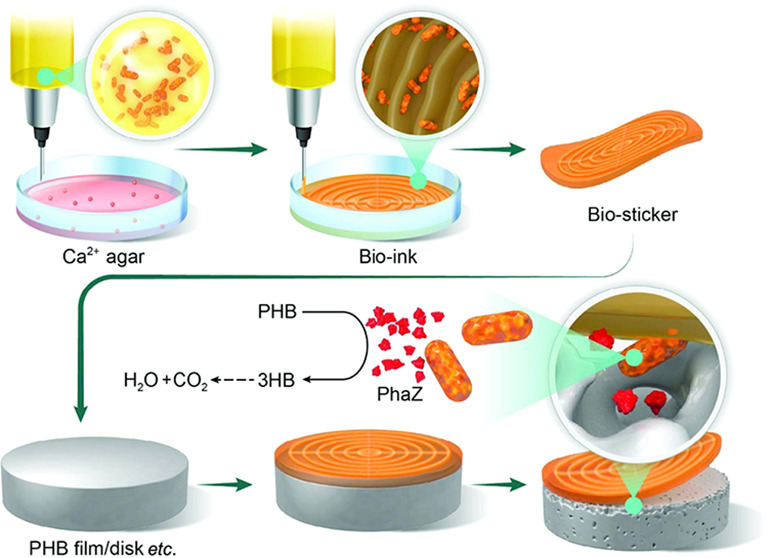

## Introduction

The rapid escalation of marine plastic pollution presents a critical threat to both marine ecosystems and coastal economies. Plastic waste has been discovered in nearly all marine ecosystems, ranging from surface waters to the deepest trenches.^1,2^ An estimated 5–13 million metric tons of land-based plastic waste enter the ocean on a yearly basis, an amount predicted to increase sharply in upcoming years.^3^ Current projections estimate that the oceans will contain more plastic than fish by weight by 2050, if the rate of plastic buildup in the ocean continues unabated.^4^ Microplastics, small plastic particles under 5 mm, are present in the ocean at up to thousands of particles per cubic meter,^5^ causing an array of toxic effects on marine animals, including cytotoxicity, genotoxicity, and growth inhibition.^6^ Ocean microplastics bioaccumulate in the tissues of marine organisms and can potentially be transmitted to humans through the consumption of contaminated organisms, causing adverse health effects.^7^

Bioplastics are a diverse category of materials that are either biobased (made of biologically grown monomers or polymers), biodegradable (able to be broken down microbially), or both. These materials have attracted attention as potential mitigating solutions to plastic waste and are a rapidly growing sector of the plastic market. However, bioplastics currently comprise less than 1% of the plastics industry.^8^ While bioplastics such as polylactic acid (PLA) have been designed and tested for disposal in industrial composting facilities, these “biodegradable” bioplastics show limited biodegradation in ocean environments.^9^ As a result, sustainable materials with demonstrated biodegradability under marine conditions are urgently needed to preserve marine ecosystems.

Polyhydroxyalkanoates (PHA) are a highly promising class of bioplastics since they share similar mechanical properties to traditional rigid structural plastics such as polypropylene.^10^ PHAs are polyesters that are naturally synthesized and accumulated within bacteria cells in order to store energy and carbon resources,^11^ and their specific chemical structure and material properties can vary depending on the specific producer organism and environmental conditions. Poly-3-hydroxybutyrate (PHB) is one of the most common types of PHA and is produced by a variety of bacteria species.^12,13^ PHB is typically considered biocompatible and suitable for use within medical implants,^14^ and marine toxicity studies have shown no measurable negative effects of PHB microparticles on survival or mobility of the small crustacean*Daphnia magna*.^15^ PHB nanosized particles also caused low short-term toxicity to*Hydra viridissima*cnidarians, though longer-term exposure was associated with increased energetic demands,^16^ and certain PHB leachates can cause acute marine toxicity,^17^ showing a need for alternative plasticizer approaches. In contrast to traditional plastics, PHB is biodegradable in ocean environments and can be broken down to H_2_O and CO_2_ without leaving behind microplastic waste.^12^ PHB biodegradation is accomplished by a variety of environmental microbes that express PHB depolymerizing enzymes, and it can be degraded in both aerobic and anaerobic environments.^18^ Importantly, PHB is able to be biodegraded in saltwater environments.^19,20^ The development of technologies that allow for accelerated or tunable PHB degradation rates in marine environments could enable this bioplastic to be employed in a diverse range of aquatic applications.

To facilitate the rapid degradation of PHB bioplastics in marine environments, this work has developed engineered living materials (ELMs) designed for the targeted and controlled degradation of plastic waste. ELMs are a class of next-generation functional materials composed of engineered biological systems that create, modify, or maintain their own material structures or properties.^21,22^ ELMs have recently been employed in the field of sustainable bioplastics in various ways, including to create living plastics that feature compostability,^23^ controllable mechanical stiffness,^24^ or triggerable depolymerization.^25^ This work utilizes 3D-bioprinting technology to design three-dimensional ELM structures with PHB depolymerization ability. A custom-built 3D bioprinter was utilized in which a commercially available, fused deposition modeling 3D printer was modified to control a syringe pump that can deposit a specialized “bio-ink” composed of alginate, which is a biocompatible hydrogel matrix, and living microbes.^26,27^ Upon extrusion onto a surface containing chemical cross-linkers, the bio-ink undergoes alginate cross-linking, resulting in a solid, three-dimensionally patterned hydrogel structure that contains embedded bacterial cells. The hypothesis was that the incorporation of microbes derived from ocean ecosystems would create living materials that can retain their viability and metabolic activities in saltwater environments. A number of marine microorganisms have been identified that are able to produce exodepolymerase enzymes active against PHB plastics.^28–32^ Among these is *Bacillus* sp. NRRL B-14911,^31^ a bacterium that secretes the PhaZ enzyme. PhaZ can break down long polymeric PHB chains into 3HB monomers, which the microbe can subsequently metabolize.^33^ Incorporation of *Bacillus* sp. NRRL B-14911 into 3D-bioprinted materials could allow for automated production of living “bio-stickers” that support spatially distributed degradation of PHB plastics throughout an extended material lifetime.

In this work, several strains of marine microbes were screened to select a strain with viability and efficient PHB depolymerization ability within polymerized bioink alginate matrices in seawater-mimicking media. 3D-bioprinted *Bacillus* sp. NRRL B-14911 resulted in biostickers with tunable PHB degradation rates that were able to maintain PHB depolymerization activity after being transferred to fresh PHB substrates. From an application standpoint, the biostickers could be attached just prior to deployment to disposable or single-use marine instruments and objects, which can have short operational lifetimes ranging from hours to several weeks.^34^ Since environmental PHB depolymerizing microbes may be sparse in specific target locations, biosticker application would increase the local concentration of biodegrading microbes, thereby allowing faster biodegradation of such devices after their operational lifetimes and reducing the lifespan of plastic waste in their target marine environments. These 3D-bioprinted living biostickers address the challenge of biodegradable plastics in marine environments and could open avenues for integrating eco-friendly, sustainable materials into the blue economy.

## Experimental (Materials and Methods)

### Bacteria Strains and Culturing Conditions

The bacteria strains used in this study are *Bacillus* sp. NRRL B-14911 ^33,35^ (Agricultural Research Service Culture Collection, United States Department of Agriculture),*Comamonas testosteroni* ^36^ (ATCC 11996), *Marinobacter* sp. NK-1 ^37^ (ATCC 700491), *Microbulbifer* sp. SOL66 ^38^ (ATCC 70072), and*Escherichia coli* BL21 (ATCC BAA-1025). Cultures of bacteria were grown in Marine Broth 2216 medium (Merck KGaA, Darmstadt, Germany) under continuous shaking at 220 rpm at 30 °C to an optical density (O.D._600_) of 1.0, except for*E. coli*, which was grown in Lysogeny broth (LB) media (Sigma-Aldrich). A 50 mL aliquot of the cultures was centrifuged at 1500 rpm for 20 min, and the resulting bacterial pellet was resuspended in either bio-ink (4% alginate (w/v) in Marine Broth) or Marine Broth, except for*E. coli*, which was resuspended in bio-ink composed of 4% alginate (w/v) in LB. Resuspended cultures were incubated at 30 °C with shaking for 48 h. Samples removed at 0 and 48 h were subjected to colony-forming unit (CFU) assays.

### Growth Curves

Cells were cultured as described above with the exception that cultures were grown in a 96-well plate. The plate was incubated at 30 °C with continuous orbital shaking at 220 rpm in a microplate reader (BioTek Synergy H1), which recorded O.D._600_ measurements every 15 min for a total of 24 or 48 h.

### Colony-Forming Unit Assays

For liquid cultures, cells were resuspended to an O.D._600_ of 0.1 in Marine Broth and were serially diluted in 1× PBS. For biostickers, the biosticker was dissolved in an equal volume of 0.1 M CaCl_2_, and resuspended cells were serially diluted in 1× PBS. From each dilution, a 100 μL aliquot was plated onto Marine Broth-agar plates. Plates were incubated overnight at 30 °C, and colonies were counted the following morning. The following equation was used to calculate the CFU/mL of the samples: (number of colonies × dilution factor)/volume of cells plated.

### 3D Bioprinting of Biostickers

Biostickers consisted of alginate hydrogels containing embedded microbes, which were ionically cross-linked to maintain shape integrity by exposure to Ca^2+^ ions upon 3D-bioprinting extrusion. Bio-inks for 3D-bioprinting were prepared by growing an overnight culture of *Bacillus* sp. NRRL B-14911 in Marine Broth at 30 °C, harvesting the cells by centrifugation at 1500 rpm for 20 min, and resuspending the cells to an O.D._600_ of 10 with a sterile solution of sodium alginate [4% (w/v)] in Marine Broth, unless otherwise specified. Bio-ink was loaded into a sterile syringe and attached to a custom-built 3D bioprinter.^26,39^ Digital models were created by using computer-aided design (CAD) software (Autodesk Fusion 360), which was then sliced using slicing software (Simplify3D). 3D-bioprinting was carried out using a print speed of 1–5 mm/s, with bio-ink extrusion rate adjusted to achieve a layer height of 0.25 mm. Unless otherwise specified, biostickers were printed using bio-ink with O.D._600_ of 10 in 4% alginate-Marine Broth, to create a biosticker with a diameter of 10 mm and a height of 2 mm, printed onto Marine Broth-PHB agar plates (50% Marine Broth, 0.5% PHB (Mango Materials, USA), and 0.3 M CaCl_2_). The G-code 3D-bioprinting instructions and the STL file that describes the three-dimensional surface geometry for the biostickers have been deposited on GitHub (https://github.com/annesmeyer/3D-bioprinted-bio-stickers). All biostickers were applied to target PHB discs or Marine Broth-PHB-CaCl_2_ agar plates in air environments unless specified.

### Clear Zone Assays of PHB Degradation

Clear zone assays were performed using the same methodology for both liquid bacterial cultures and for biostickers. Liquid bacterial culture was pipetted onto Marine Broth-PHB-CaCl_2_ agar plates, or biostickers were 3D-bioprinted onto Marine Broth-PHB-CaCl_2_ agar plates. After solidification, the biostickers were transferred to fresh plates if needed using a sterile spatula. Plates were incubated at 30 °C, and PHB degradation was monitored on a daily basis by acquiring scanned images of the plates from underneath. ImageJ software^40^ was used to quantify clear zones from the scanned images by (i) setting the image scale via the 10 mm scale bar; (ii) using threshold-based edge detection to delineate the boundary of the clear region; and (iii) recording radius (*r*) of the clear zone as the perpendicular distance from the edge of the bacteria colony/biosticker to the boundary of the clear region. Clear zone values represent the average of four biological replicates (*n* = 4).

The mass of degraded PHB in clear zone assays was calculated as follows: each plate was assumed to contain approximately the same agar volume (20 mL) and PHB loading (0.5% w/v = 0.005 g mL^–1^). PHB powder was assumed to be approximately uniformly dispersed through an agar layer of constant thickness.

Thus, the total PHB mass per plate

$$m_{\mathrm{total}}=\mathrm{concentration}\times \mathrm{volume}=0.005\;g/\mathrm{mL}\times 20\;\mathrm{mL}=0.10\;g$$

From the scanned plate image, ImageJ software was used to measure the plate radius (*R*) and clear zone radius (*r_t_*). The assumption of constant agar layer thickness implies a constant mass of PHB degraded per measured area of clear zone.

To convert the clear zone area to PHB mass

$$m_{\mathrm{degraded}}\left(t\right)=m_{\mathrm{total}}\times \frac{\pi \left(r_{t}^{2}-r_{0}^{2}\right)}{\pi R^{2}}=m_{\mathrm{total}}\times \frac{\left(r_{t}^{2}-r_{0}^{2}\right)}{R^{2}}$$

### SEM Imaging

Biostickers were applied to degrade PHB discs (1 mm thickness, 44.5 mm diameter, Mango Materials, USA) over 28 days at 30 °C. Postincubation, the biostickers were removed from the PHB films, which were cleansed and freeze-dried in preparation for SEM analysis. The films were sputter-coated with approximately 5 mm gold film at 20 mA for 60 s and positioned onto SEM stubs. SEM imaging was performed on a JEOL JSM-IT500HR InTouchScope scanning electron microscope at an accelerating voltage of 5 kV, and backscattered electron images were captured at multiple magnifications.

### Mass Loss Analysis of PHB Discs

For incubation in air, PHB discs (1 mm thickness, 44.5 mm diameter) were sterilized with ethanol, and each disc was fully covered by a biosticker containing *Bacillus* sp. NRRL B-14911,*E. coli* BL21, or hydrated with sterile artificial seawater. The discs were statically incubated at 30 °C. Postincubation discs were removed from the biosticker, cleaned, and weighed to determine mass loss at 7-day intervals.

To measure the pH of biostickers incubated on solid PHB in air, biostickers consisting of alginate only (negative control) or alginate containing *Bacillus* sp. NRRL B-14911 were incubated on a PHB sheet for 5 weeks at 30 °C. pH measurements were taken every 2–3 days for the first 3 weeks and once a week thereafter using MQuant pH-indicator strips. Biostickers were rehydrated approximately 1 h prior to each pH measurement with 500 μL of Milli-Q H_2_O to allow for equilibration and to provide enough moisture for the pH to be measured.

For incubation in artificial seawater, biostickers were transferred to a premassed ∼4 g PHB sheet with dimensions of 35 cm × 0.2 cm × 40 cm and incubated to adhere overnight at 30 °C. PHB sheets with or without an adhered biosticker were placed in 250 mL of sterile artificial seawater (423 mM NaCl, 9 mM KCl, 12.3 mM CaCl_2_, 23 mM MgCl_2_, 25 mM MgSO_4_, 2.14 mM NaHCO_3_) in a 1 L beaker and incubated at 30 °C. The beaker was shaken 5–10 times per week to facilitate aeration and mimic ocean conditions. Every week, 1 mL of artificial seawater was removed from the incubating beaker and used to perform a colony-forming unit assay in triplicate, as described above. After 7 weeks of incubation, the PHB discs were removed from the artificial seawater, cleaned with 70% EtOH, and incubated in a 65 °C oven overnight prior to measuring the final mass.

### Rheology Testing

Biostickers were prepared with or without *Bacillus* sp. NRRL B-14911 and were incubated on CaCl_2_-Marine Broth-agar plates at 4 or 30 °C for 21 days. Shear rheology was performed using a TA HR-30 rheometer, using a 20 mm parallel plate geometry on a steel Peltier plate to ensure a uniform shear field during testing. Strain sweep tests were performed at a frequency of 1 Hz on a range from 0.01 to 100% strain to determine the limit of the linear viscoelastic (LVE) range and to identify the material’s yield point. The dynamic moduli were then analyzed, specifically the storage modulus (*G*′) and loss modulus (*G*″).

### Tensile Testing

Biostickers were 3D-bioprinted with or without *Bacillus* sp. NRRL B-14911 into the ASTM D638-05 standard dog-bone shape,^41^ with an average sample thickness of 1.528 mm (ranging from 0.724 to 2.047 mm). Samples were incubated on CaCl_2_-Marine Broth-agar plates at 4 or 30 °C for 21 days. The samples were washed with a 0.1 M CaCl_2_ solution prior to removal. Quasi-static tensile testing was performed via monotonic tensile loading to failure on a universal testing machine (Zwick-Roell Z010) with a 20 N load cell (X-Force HP, Zwick-Roell), at a displacement speed of 1.1 mm·s^–1^. Nonwoven fabrics were applied to reduce specimen slippage in the grips. Specimen width and thickness were measured optically, and the gauge length was measured with calipers. Engineering strain was calculated as crosshead displacement divided by the initial gauge length. Engineering stress was calculated as the force divided by the cross-sectional area. Elastic modulus was calculated by fitting a linear regression to the range from 0 to 0.15 strain (mm/mm) of the stress–strain curve and selecting the maximum value. Ultimate stress was reported based on the maximum stress prior to failure for samples that broke in the center section.

### Adhesion Testing

Adhesive properties of the biostickers were tested via a protocol inspired via lap shear testing protocols.^42^ Rectangular biostickers (*L* × *W* × *H* = 47.22 mm × 12.65 mm × 3.71 mm) were 3D-bioprinted using 4% alginate bio-ink with or without *Bacillus* sp. NRRL B-14911 onto CaCl_2_-Marine Broth-agar plates and incubated at 4 or 30 °C for 21 days. PHB sheets (thickness 1.99 mm, Goodfellow U.K.) were gripped in a vertical fixed position within a universal testing machine (Zwick-Roell Z010) with a 20 N load cell. The biostickers were tightened in the top grip, and the surface was patted dry. The biostickers were pressed into contact with the PHB sheet with an overlap distance of 18.87 mm. The biosticker specimens were pulled at a constant crosshead displacement rate of 2 mm·s^–1^. Interfacial adhesion strength was reported by dividing the maximum force by the initial area of contact between the PHB sheet and biosticker specimen. The energy dissipated was calculated by integrating the area under the force–displacement curve.

## Results

The goal of this project was to develop PHB-degrading biostickers by patterning a bio-ink mixture of living microbes and alginate polymer in three dimensions onto a surface supplemented with calcium ions (Ca^2+^) (Figure 1). The Ca^2+^ ions promote cross-linking of the alginate chains, solidifying the bio-ink into a flexible, adhesive biosticker. The biosticker should provide mechanical stability as well as a hydrated environment for the growth and function of PHB-degrading marine microbes, allowing them to depolymerize PHB materials over extended time periods.

**Figure 1:**
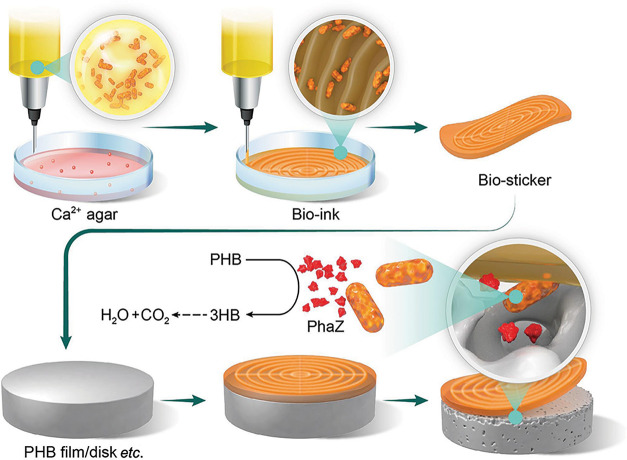
3D bioprinting of marine bacteria for the biodegradation of bioplastics. Bio-ink consisting of marine bacteria and alginate is patterned in 3D onto an agar surface containing Ca^2+^. Interaction of bio-ink with Ca^2+^ ions drives the cross-linking of alginate chains to form a solid biosticker that can be transferred onto a PHB bioplastic object. The embedded microbes produce and excrete the PhaZ enzyme to catalyze the biodegradation of PHB polymers into 3HB monomers, which are further metabolically decomposed into H_2_O and CO_2_.

### Design of 3D-Bioprinted Biostickers with PHB Degradation Activity

Bacteria strains were screened for their ability to degrade PHB in marine-like environments. Four bacteria strains were selected that were isolated from marine sources and had been observed to demonstrate PHB depolymerization ability: *Bacillus* sp. NRRL B-14911,^33,35^ *C. testosteroni*,^36^ *Marinobacter* sp. NK-1,^37^ and *Microbulbifer* sp. SOL66.^38^ Growth curves recorded over a 24-h incubation at 30 °C in Marine Broth, which mimics the salinity and mineral content of seawater,^43,44^ indicated that all four strains were able to proliferate in ocean-mimicking conditions (Figure 2A).

**Figure 2:**
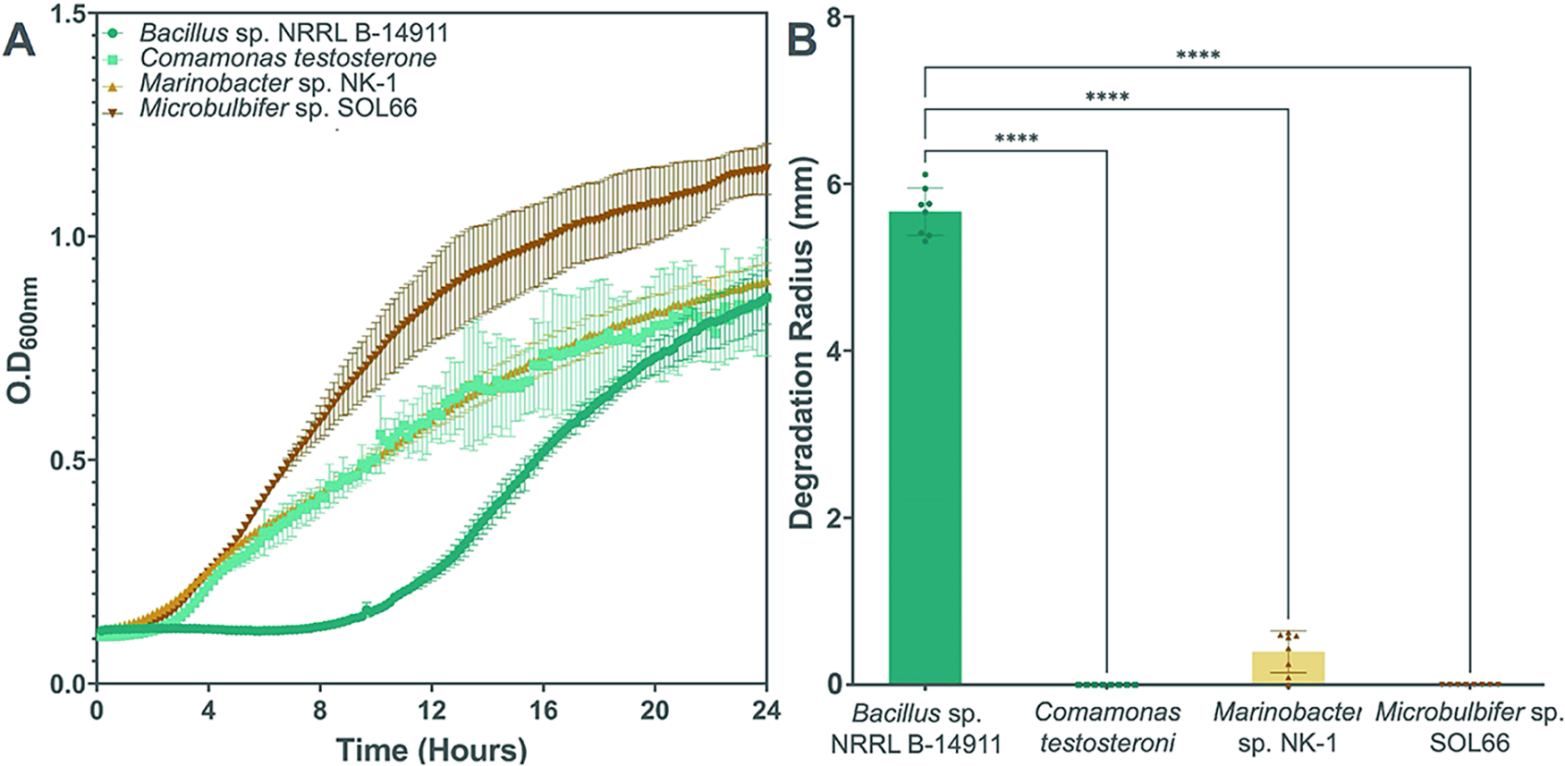
Growth and PHB degradation of marine bacteria. (A) Growth curves of *Bacillus* sp. NRRL B-14911 (dark green), *C. testosteroni* (light green), *Marinobacter* sp. NK-1 (light brown), and *Microbulbifer* sp. SOL66 (dark brown) cultured in Marine Broth at 30 °C. (B) Degradation of PHB by the marine bacteria species was determined by growing bacterial colonies on Marine Broth-agar plates containing PHB powder and measuring the radii of the clear zones of depolymerized PHB formed around the colonies after 7 days (*n* = 8). ****P ≤ 0.0001 by one-way ANOVA statistical analysis.

To measure the ability of these strains to degrade PHB, cultures of each of the strains were grown on a Marine Broth-agar plate containing powdered PHB. A clear zone assay was employed to measure the PHB depolymerizing activity of these bacterial colonies. Depolymerization of the opaque PHB creates a clear zone within the PHB plate, the radius of which can expand over time at different rates correlating to depolymerization rates (see Figure S1A for an example clear zone assay performed with 3D-printed bacteria).^45^ Among the marine bacteria tested, only *Marinobacter* sp. NK-1 and *Bacillus* sp. NRRL B-14911 created measurable clear zones of PHB degradation after 7 days, and the clear zones created by the *Bacillus* sp. NRRL B-14911 colonies were significantly larger than those observed for the other strains (Figures 2B and S1B). Due to its higher rate of PHB degradation on ocean-mimicking media, *Bacillus* sp. NRRL B-14911 was used throughout the rest of this work.

Next, the growth of *Bacillus* sp. NRRL B-14911 was evaluated in a variety of marine-like culturing conditions. Growth curves of *Bacillus* sp. NRRL B-14911 in Marine Broth at a range of temperatures indicated robust growth of this bacterium at both 30 and 25 °C, with somewhat slower growth and lower carrying capacity at 20 and 18 °C (Figure S2A). The salt tolerance of *Bacillus* sp. NRRL B-14911 was investigated by measuring the growth curves of the strain in liquid growth media with a range of concentrations of added sodium chloride. Robust growth was observed for sodium chloride concentrations ranging from 3 to 7%, with moderate growth inhibition at 1% sodium chloride (Figure S2B). These results indicate that *Bacillus* sp. NRRL B-14911 shows viability and strong growth in temperatures corresponding to the warmer average surface ocean temperatures, as well as in salinities corresponding to the mean ocean salinity, which is 3.5%.^46^

To determine whether 3D-bioprinting parameters including CaCl_2_ cross-linker and alginate bio-ink can affect *Bacillus* sp. NRRL B-14911, its growth and viability were tested under a range of 3D-bioprinting conditions. Ca^2+^ concentration can be varied to tune the degree of cross-linking of alginate-based hydrogels.^47^ Growth curves of *Bacillus* sp. NRRL B-14911 in Marine Broth containing a range of supplemental CaCl_2_ concentrations indicated that bacterial growth could be observed at concentrations between 0.01 and 0.10 M CaCl_2_ (Figure S2C). Similarly, *Bacillus* sp. NRRL B-14911 bioprinted onto Marine Broth-agar surfaces containing a range of CaCl_2_ concentrations and incubated for 7 days showed no significant change in viability at concentrations from 0.01 to 0.10 M CaCl_2_ (Figure S2D), well beyond the typical range used for alginate cross-linking in bioprinting applications.^26,39,48–50^ To test for any effect on cell viability resulting from the presence of alginate or cell entrapment within a hydrogel matrix, samples were cultured in Marine Broth under identical conditions with the exception of the addition of alginate (for liquid bio-ink samples) or the addition of both alginate and Ca^2+^ cross-linker (for polymerized bio-ink samples). The viability of *Bacillus* sp. NRRL B-14911 in liquid Marine Broth, measured by colony-forming units (CFUs), was steady for the first 4 days of incubation but was observed to decrease by ∼4 orders of magnitude after 5 days of incubation (Figure S2E). In contrast, the incubation of *Bacillus* sp. NRRL B-14911 in either liquid or polymerized bio-ink resulted in constant high levels of viability over 28 days of incubation (Figure S2E), indicating a protective effect of alginate on bacterial viability. Logistic growth curve modeling of CFU viability data from 3D-bioprinted *Bacillus* sp. NRRL B-14911 predicted a stable plateau phase for 3D-bioprinted bacteria (Figure S2F), suggesting longer-term viability of biomass within biostickers that could support extended PHB biodegradation.

### Degradation of PHB Sheets by Biostickers

To characterize the PHB degradation activity of the biostickers, *Bacillus* sp. NRRL B-14911 was 3D-printed into biostickers that were placed overtop of 1-mm-thick PHB sheets to fully cover them, followed by incubation in air for 28 days at 30 °C (Figure S3). The biostickers were printed into 2-mm-thick, 10-mm-diameter circular slabs throughout this work, except where noted, a geometry that was designed to provide a wide surface for enhanced adhesion to and interaction with target objects, with a short height to promote fluid and/or gas exchange with the environment. Negative control PHB sheets were incubated with 3D-printed alginate samples that did not contain bacteria. Postincubation, the biostickers were removed, and the cleaned PHB sheets were analyzed by scanning electron microscopy (SEM). The micrographs of the PHB incubated with *Bacillus* sp. NRRL B-14911 biostickers showed a corroded morphology characterized by visible pitting, surface irregularities, and microscale roughness that was fairly uniform across the discs (Figure 3A,B). In contrast, the micrographs of the control PHB samples incubated with only 3D-printed alginate showed a low surface roughness with little pitting (Figure 3C,D).

**Figure 3:**
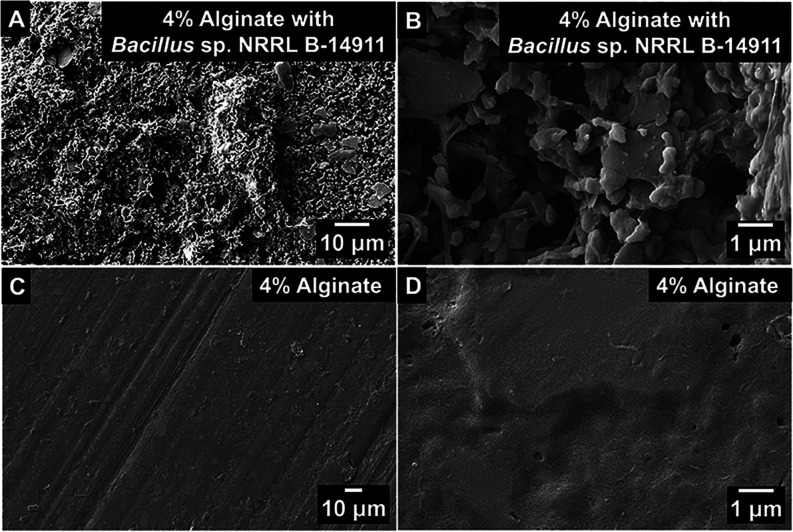
Biodegradation of solid PHB sheets by *Bacillus* sp. NRRL B-14911 biostickers. (A, B) SEM images of PHB sheets incubated with *Bacillus* sp. NRRL B-14911 biosticker for 28 days. (C, D) SEM images of PHB sheets incubated with biostickers not containing bacteria for 28 days. Biostickers and bacteria were removed prior to imaging. Micrographs are representative of samples obtained from 3 biorepeats containing 5 samples each, imaged at multiple central locations on the PHB sheets that were fully covered by biostickers.

To quantify the degradation of solid PHB sheets by biostickers over time, mass loss tests were performed. PHB sheets of 1 mm thickness were cut into discs and UV-sterilized, and each disc was fully covered by a biosticker containing *Bacillus* sp. NRRL B-14911. Control discs were covered either by biostickers containing*E. coli* BL21, which is a bacteria strain not known to degrade PHB, or by cell-free biostickers hydrated with sterile artificial seawater. The discs were incubated statically at 30 °C, and their masses were measured weekly over a 28-day period. Significant losses in mass were observed during the incubation period for the PHB discs incubated with *Bacillus* sp. NRRL B-14911 biostickers, which lost 6.3 ± 0.4% of their initial mass after 28 days of incubation (Figure 4). Both types of control discs demonstrated a net gain of mass.*E. coli* biostickers resulted in a gain of 3.9 ± 0.3%, and seawater biostickers resulted in a gain of 1.8 ± 0.2% after 28 days, consistent with water uptake and/or bacterial or alginate adherence. A one-way ANOVA followed by Tukey’s posthoc test showed a highly significant difference between the *Bacillus* group and either control (*P* ≤ 0.0001). These results indicate that 3D-printed *Bacillus* sp. NRRL B-14911 is able to biodegrade solid PHB materials over the course of weeks.

**Figure 4:**
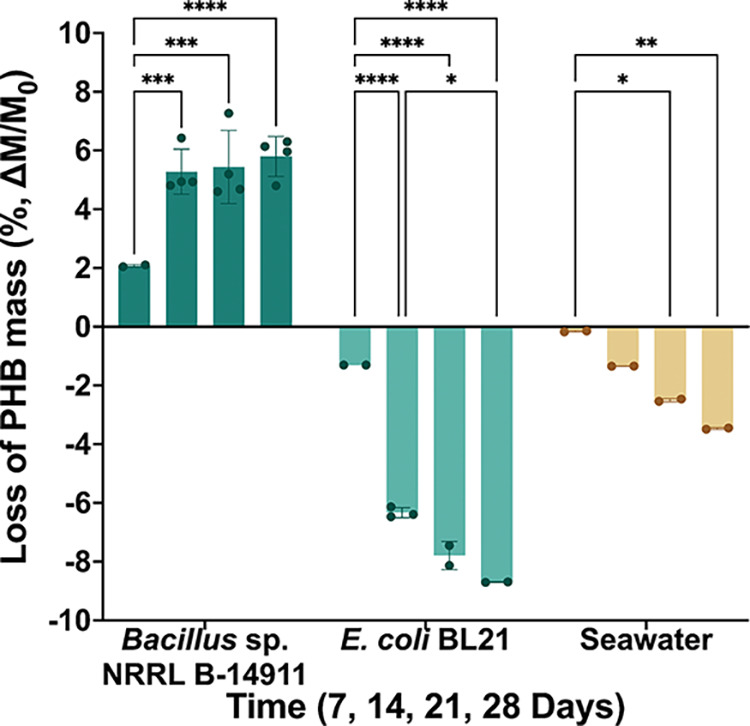
Biodegradation of solid PHB sheets over several weeks by *Bacillus* sp. NRRL B-14911 biostickers. Mass loss of PHB discs incubated for 7, 14, 21, and 28 days at 30 °C with a biosticker containing *Bacillus* sp. NRRL B-14911,*E. coli* BL21, or sterile seawater (*n* = 4). *P ≤ 0.05, **P ≤ 0.01, ***P ≤ 0.001, and ****P ≤ 0.0001 by one-way ANOVA statistical analysis.

Interestingly, while solid PHB discs incubated with biostickers showed an overall monotonic decrease in mass with longer incubation times, a larger decrease in mass was measured between 7- and 14-day samples than between 14- and 21-day or 21- and 28-day samples (Figure 4). These data suggest that PHB degradation could slow or become limited over time under these experimental conditions by factors such as limited nutrient availability or buildup of inhibitory bacterial waste products, which would be expected to be less prevalent under natural ocean conditions with higher water circulation. Measurement of the pH of biostickers incubated on solid PHB indicated that the pH remained within 1 pH unit, between pH 6 and 7, throughout the entirety of the 5-week incubation, with the initial and final pH readings within 0.25 units of each other for both alginate-only and *Bacillus* NRRL B-14911-containing biostickers (Figure S4). These data suggest that pH effects on microbial physiology or enzyme activity may not play a major role in the PHB degradation kinetics over extended incubations.

To measure the ability of the biostickers to degrade solid PHB while submerged in an ocean-mimicking liquid, biostickers were adhered to solid PHB discs that were placed into artificial seawater. Negative control samples did not receive biostickers. Samples were incubated for 7 weeks at 30 °C with periodic shaking. To measure the concentration of viable *Bacillus* sp. NRRL B-14911 microbes within the artificial seawater over time, artificial seawater samples were removed every week, and CFU/mL values were determined. *Bacillus* sp. NRRL B-14911 was found to remain viable with no significant change in viability throughout the entire 7-week incubation for the samples containing biostickers, while no measurable viable bacteria were detected for the negative control samples (Figure 5A). Mass loss analysis of the PHB sheets was performed by comparing the weights of the PHB sheets prior to and after the 7-week incubation in artificial seawater. A single-tailed bootstrap *t*-test indicated that the PHB sheet with the biosticker exhibited significantly greater mass loss than the sheet without the biosticker (Figure 5B), indicating that the biostickers can accelerate the degradation of PHB objects both in air and liquid environments. The PHB mass loss under liquid conditions could potentially have been increased with increased surface contact between the biosticker and the PHB object or with periodic refreshing of the artificial saltwater medium, which would have reduced any byproduct accumulation. Lower mass loss percentages were observed for the PHB samples incubated with biostickers in liquid artificial seawater (Figure 5) compared to samples incubated in air (Figure 4), despite the longer incubation times for the samples in artificial seawater, potentially due to decreased expression or increased dilution of microbially produced PhaZ enzyme in the liquid environments.

**Figure 5:**
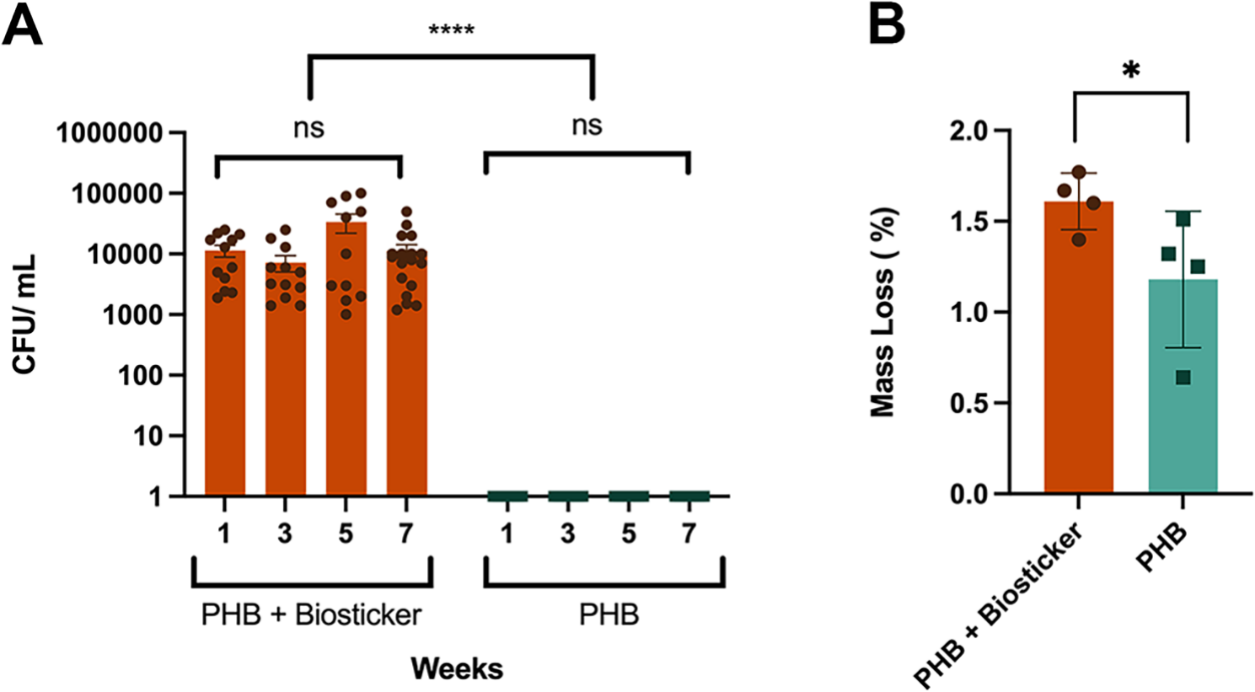
Biodegradation of solid PHB sheets submerged in artificial seawater by biostickers. (A) CFU/mL over time for artificial seawater containing PHB discs incubated for 7 weeks at 30 °C, with or without a biosticker containing *Bacillus* sp. NRRL B-14911. (B) Mass loss of PHB discs submerged in artificial seawater and incubated for 7 weeks at 30 °C, with or without a biosticker containing *Bacillus* sp. NRRL B-14911 (*n* = 4). *P ≤ 0.05 by single-tailed bootstrap *t*-test analysis.

### Tuning of PHB Degradation by Biostickers

To obtain quantitative data on the kinetics of PHB degradation by biostickers, clear zone assays were performed. Clear zone experiments are an established and straightforward assay to reproducibly demonstrate baseline PHB degradation rates under controlled conditions,^33,45,51–53^ allowing for the direct comparison of the effects of tuning different biosticker parameters. Biostickers were incubated at 30 °C on Marine Broth-agar plates containing PHB powder. By day 4 of incubation, the regions directly beneath the biosticker had been cleared of PHB, and the clear zones continued to expand over 28 days of incubation (Figure 6A). Clear zones within PHB agar plates were created by biostickers with a variety of shapes (Figures 6A and S5), indicating that PHB biodegradation can be carried out by biostickers with a range of geometries. Biosticker geometry may influence PHB degradation kinetics since the rate of diffusion of PhaZ enzyme would presumably be highest from biostickers with a higher surface-to-volume ratio; further experimentation would be able to measure any effects systematically.

**Figure 6:**
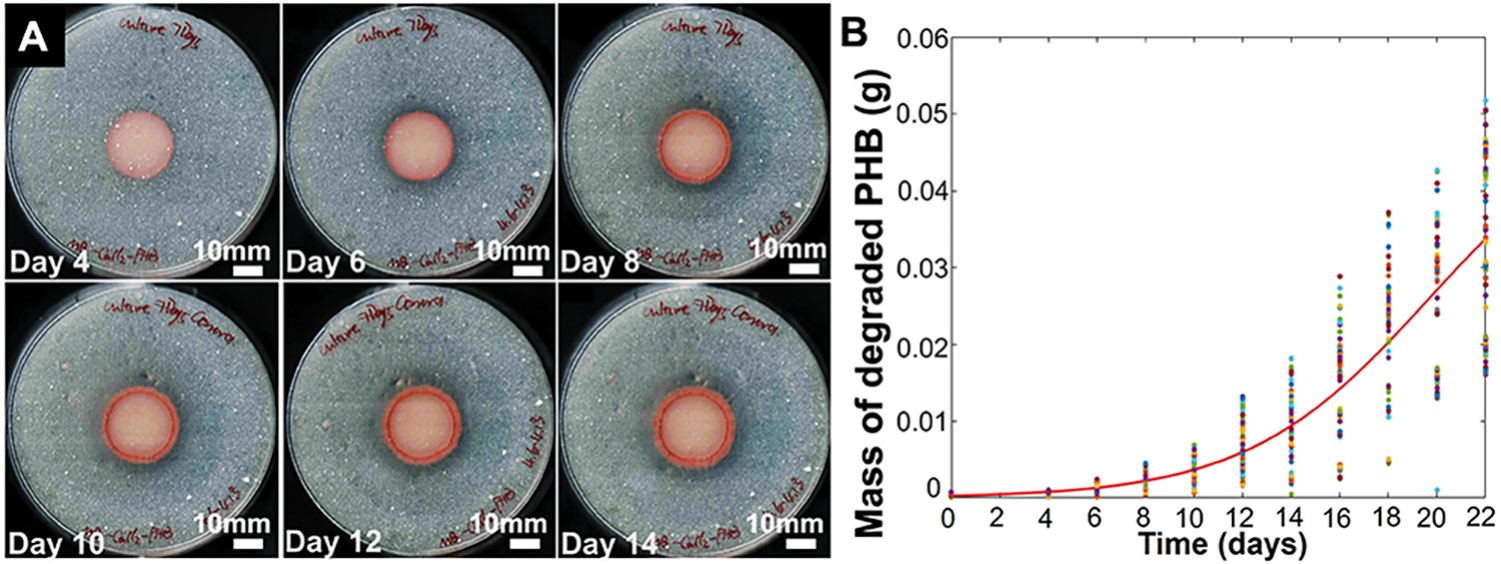
Progression of PHB degradation by biostickers. (A) Clear zone assay for biostickers incubated on Marine Broth-PHB agar plates. Samples were incubated at 30 °C for 28 days (*n* = 4). (B) The mass of PHB degraded by biostickers over 22 days of incubation was calculated and fit to a logistic equation.

Logistic modeling of PHB degradation by biostickers over time indicated that the rate of PHB degradation increased over the first 2–3 weeks of incubation (Figure 6B), predicting sustained and robust PHB degradation ability of biostickers over an extended period of time following application. This analysis was performed for 22 days of incubation due to the complete consumption of the PHB powder in the plates at later time points. The more sustained PHB degradation observed in these experiments, compared to the mass loss experiments in Figure 4, may be due to the PHB being in powder form, where the smaller particle size can result in more accurate degradation data,^54,55^ as well as the greater ability for secreted PhaZ enzyme to diffuse through the agar plates. To compare the PHB degradation performance of 3D-bioprinted bacteria relative to unencapsulated cells, clear zone assays were performed using either a standard biosticker or an equivalent volume of *Bacillus* sp. NRRL B-14911 liquid culture applied at one of three different cell densities. The radii of the clear zones over time were similar for the biostickers and for all three concentrations of unencapsulated cells (Figure S6), indicating that alginate encapsulation does not have a measurable impact on PHB degradation performance.

To determine how the PHB biodegradation rate of the biostickers can be tuned, clear zone assays were performed while individually altering several of the experimental parameters. To determine the effect of changing PHB concentration, biostickers were 3D-bioprinted onto Marine Broth-agar plates containing PHB at concentrations ranging from 0.1 to 2.0% and incubated at 30 °C for 22 days. CFU analysis of the biostickers at 7-day increments indicated that the concentration of colony-forming units within the biostickers exhibited significant decreases of roughly 1–2 logs over the 22-day incubation period for all but the lowest PHB concentration but retained a robust 10^7^ CFU/mL after 22 days for the majority of the PHB concentration conditions, including the two conditions with the highest PHB concentrations (Figure S7A). Clear zone analysis showed significantly higher rates of PHB clear zone expansion at lower PHB concentrations, indicating a negative correlation between the PHB concentration and rate of clear zone expansion (Figure 7A,B). The similar CFU values for the biostickers over time between different PHB concentrations indicated that the differences in rates of clear zone expansion were likely not due to changes in the viability of the bioprinted microbes at different PHB concentrations but could reflect changes in the concentration of PhaZ enzyme relative to PHB substrate.

**Figure 7:**
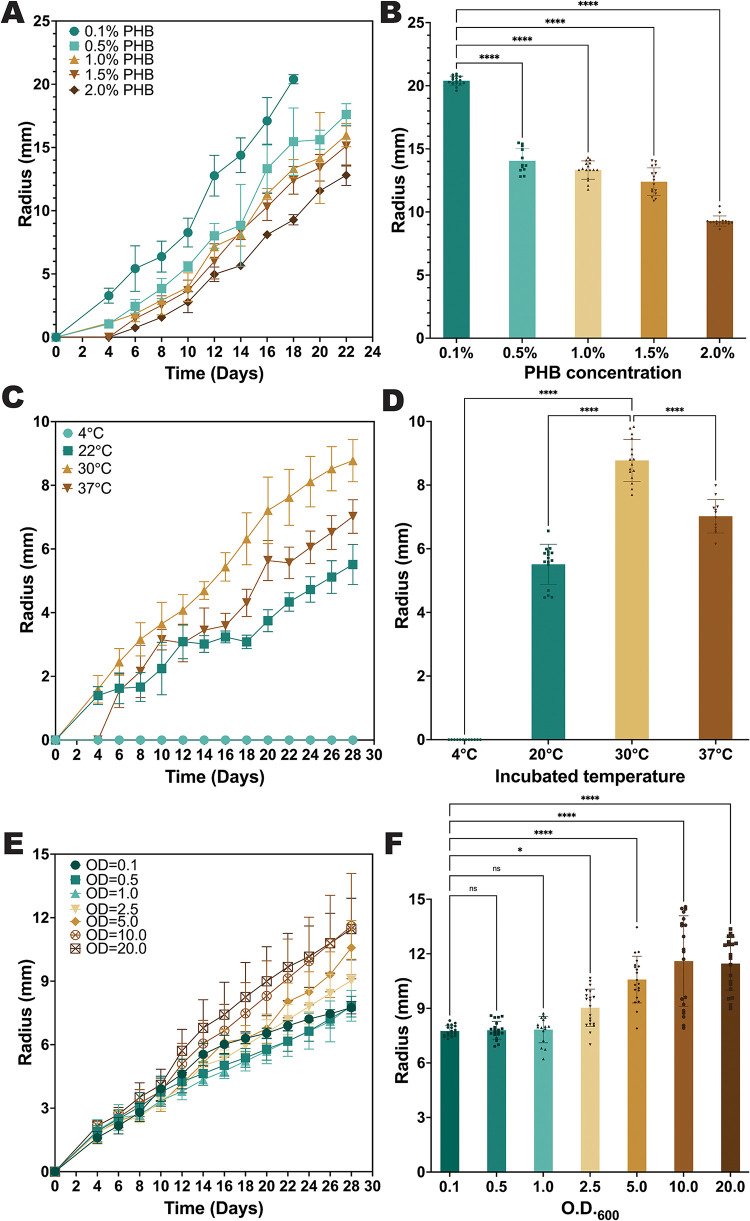
PHB degradation can be tuned by altering experimental parameters. Clear zone assays for biostickers at an O.D._600_ of 10, incubated at 30 °C on Marine Broth-agar plates containing 0.5% PHB powder, except where altered parameters are noted. (A, B) Clear zone radius over time (A) and on day 18 (B) for biostickers bioprinted onto plates containing varying PHB concentrations (*n* = 16). (C, D) Clear zone radius over time (C) and on day 28 (D) for biostickers incubated at varying temperatures (*n* = 16). (E, F) Clear zone radius over time (E) and on day 28 (F) for biostickers printed using bio-ink with varying initial O.D._600_ (*n* = 16). The time points that were analyzed in panels (B, D, F) for statistical differences between conditions were 18 (B) or 28 days (D, F). *P ≤ 0.05, ****P ≤ 0.0001, ns = not significant by one-way ANOVA statistical analysis.

To determine the effect of incubation temperature on biosticker PHB degradation efficiency, biostickers were incubated on Marine Broth-PHB agar plates at 4, 22, 30, or 37 °C for 28 days. CFU analysis of the biostickers indicated that the concentration of colony-forming units within the biostickers showed no significant changes during the 28-day incubation period at 30 or 37 °C, whereas it decreased significantly by roughly 1–2 logs at incubation temperatures of 4 or 22 °C (Figure S7B). Clear zone analysis showed that PHB degradation by the biostickers was fastest at 30 °C, significantly slower at 22 and 37 °C, and not detectable at 4 °C (Figure 7C,D). The rate of PHB degradation at 37 °C increased after the first 6 days of incubation, after which it was similar to the degradation rate at 30 °C, perhaps indicating adaptation to the higher temperature condition over time. The lack of detectable PHB degradation at 4 °C despite the viability of the cells within the biosticker indicates that the bacteria at this colder temperature remain alive but with very low or nonexistent PHB degradation activity, showing that microbial viability and PHB degradation activity are not necessarily linked. These data are consistent with previous studies that found that cooler seawater temperatures resulted in reduced specific activity for PHA depolymerizing enzymes as well as lower metabolism of marine microbes that degrade PHA bioplastics.^56,57^

To determine the effect of the concentration of initial biomass on biosticker PHB degradation efficiency, biostickers were prepared using bio-ink with cell densities varying from O.D._600_ 0.1 to O.D._600_ 20 and incubated on Marine Broth-PHB agar plates. CFU analysis of the biostickers showed that the concentration of colony-forming units within the biostickers was approximately 10^8^ CFU/mL at the 7-day time point for all samples and exhibited a consistent, gradual but significant decrease by 1–2 logs over the incubation period, regardless of initial cell density (Figure S7C). The similar CFU/mL values of the biostickers at the 7-day time point may indicate that the 3D-bioprinted bacteria were able to replicate up to a comparable carrying capacity for these specific incubation conditions during the first week after deposition. Clear zone analysis indicated that higher initial biomass corresponded to significantly greater PHB degradation over time, with the highest-O.D. bio-inks (e.g., O.D._600_ 10, 20) exhibiting clear zones with approximately 65% larger radius after 28 days compared to the lowest-O.D. bio-ink (O.D._600_ 0.1) (Figure 7E,F). These results indicate that initial cell density within biostickers enhances PHB degradation efficiency, despite the cell viability within the biostickers remaining roughly similar between conditions throughout days 7–28 of the experiment, potentially due to increased accumulation of PHB-degrading enzymes during early time periods within the experiment.

While changes in PHB concentration, incubation temperature, and initial biomass were all observed to tune the rate of PHB degradation by 3D-bioprinted biostickers, altering other 3D-bioprinting parameters had less effect on PHB degradation rate. To test the effect of biosticker hydrogel density on PHB degradation, biostickers were prepared with alginate concentrations varying from 2 to 6% and incubated on either Marine Broth-PHB agar plates or PBS–PHB agar plates. PHB degradation rates varied by less than 15% (Figure S8A,B), and CFU/mL values decreased significantly but by less than 1 log (Figure S8C) across all alginate concentrations. To test the effect of biosticker height on PHB degradation, biostickers were prepared with heights ranging from 1 to 15 mm and incubated on Marine Broth-PHB agar plates. Most biostickers of varying heights showed statistically indistinguishable differences in PHB degradation, with a modest increase for the tallest biostickers (Figure S8D,E). CFU/mL values were uniformly high across all biosticker heights, and most conditions did not show a significant change over time (Figure S8F). To test the effect of biosticker radius on PHB degradation, biostickers were prepared with radii ranging from 10 to 25 mm and incubated on Marine Broth-PHB agar plates. PHB degradation was similar across biostickers of all radii, with a significant but minor 2 mm increase in PHB degradation radius for the smallest 10 mm biosticker (Figure S8G,H) and similar high CFU/mL for the biostickers that significantly decreased by approximately 1 log across a 24-day time period (Figure S8I). These results indicate that the PHB degradation rate of the biostickers is only modestly affected by their geometry and hydrogel cross-linking density.

### Biostickers Retain PHB Degradation Activity Following Storage and Transfer

For deployment in application scenarios to degrade bioplastic objects, biostickers would likely be 3D-bioprinted and then temporarily stored or transported to end users for utilization. The biostickers would need to retain viability and PHB degradation activity for several days or weeks in order to still be able to depolymerize PHB once transferred to their target objects following a period of storage or transportation. To test this scenario, biostickers were 3D-bioprinted onto Marine Broth-PHB agar plates and stored for 7 or 14 days at 30 °C, then transferred to a fresh Marine Broth-PHB agar plate using sterile tweezers to test for PHB degradation activity using a standard clear zone assay (Figure 8A). The biostickers were able to be transferred to different plates without breaking, adhered directly to the tweezers throughout the transfer, even when inverted, and adhered directly to the new Marine Broth-PHB agar surface post-transfer (Video S1). Clear zone analysis indicated that biostickers that had been transferred after 7 or 14 days of storage showed robust PHB degradation (Figure 8B,C), accompanied by uniformly high CFU/mL for the biostickers across all conditions, with significant decreases over time of approximately 1 log (Figure 8D). Transferred biostickers showed higher PHB degradation at the earliest time points, likely due to the printed microbes having had additional time during the storage period to divide and accumulate biomass (Figure 8B). After a delayed burst of PHB degradation, the nontransferred biostickers adopted a final rate of PHB degradation that was similar to the transferred biostickers (Figure 8B), indicating that the biostickers can maintain a steady-state rate of PHB degradation for several weeks postprinting.

**Figure 8:**
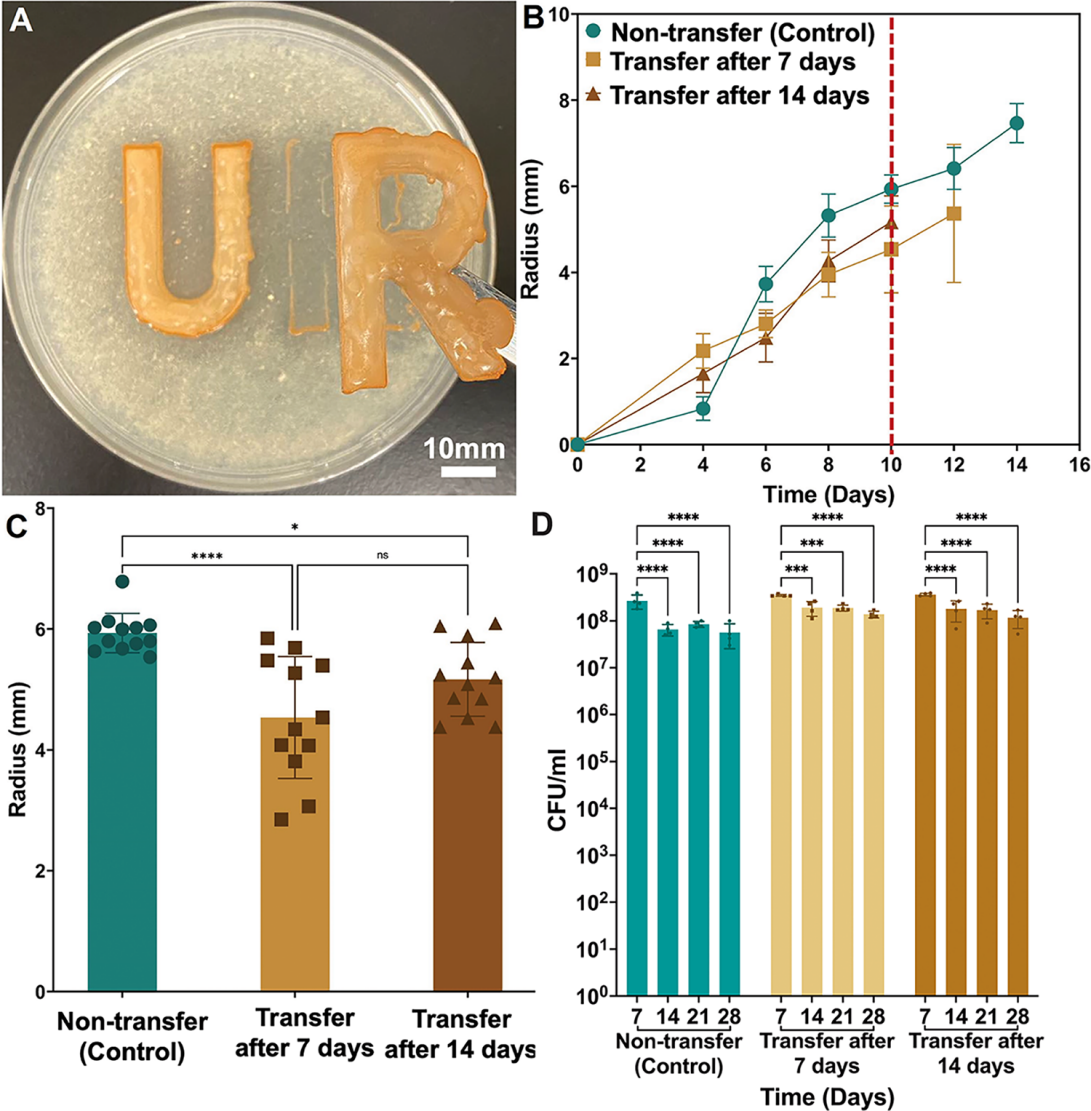
Transferred biostickers retain PHB degradation activity. (A) Biostickers can be transferred following incubation on Marine Broth-PHB agar plates. (B, C) Clear zone radius over time (B) and on day 10 (C), and CFU/mL over time (D) for biostickers incubated on Marine Broth-PHB agar plates (green) or transferred onto fresh Marine Broth-PHB agar plates following 7 (tan) or 14 (brown) days of storage (*n* = 16). Vertical dashed red line in panel (B) indicates time points that were plotted in panel (C). *P ≤ 0.05, ****P ≤ 0.0001, ns = not significant by one-way ANOVA statistical analysis.

### Mechanical Properties of Biostickers

Encapsulation of bacteria within hydrogels typically has either little immediate effect on hydrogel stiffness or causes increased stiffness.^58^ However, PHB-degrading biostickers may need to maintain their mechanical robustness over several weeks of storage prior to application to target PHB objects to allow time for shipping to end users or for preparations to deploy target PHB objects. The biostickers were therefore incubated for 21 days prior to testing their mechanical properties to assess performance after several weeks of storage in either refrigerated or warm conditions. To test the viscoelastic behavior of the 3D-bioprinted hydrogels, we performed rheology testing. Biostickers either containing *Bacillus* sp. NRRL B-14911 or not containing microbes were stored on Marine Broth-PHB agar plates for 21 days at either 4 or 30 °C, to simulate temperatures toward the higher and lower range of typical ocean water temperatures. Strain sweep oscillatory tests were performed on the biostickers to determine their storage modulus (*G*′) and loss modulus (*G*″) (Figures 9A and S9). Notably, for all sample groups, at strains less than 1%, *G*′ was consistently higher than *G*″ by approximately 1 order of magnitude, indicating that the samples exhibited predominantly elastic, gel-like behavior. The storage modulus *G*′ was approximately 10^5^ Pa for the samples stored at 4 °C, either containing or not containing microbes, as well as the samples without microbes stored at 30 °C, which is a typical value for alginate-based materials.^59,60^ In contrast, the samples containing *Bacillus* sp. NRRL B-14911 stored at 30 °C had a lower storage modulus around 1 × 10^4^ Pa (Figure 9A). The samples containing *Bacillus* sp. NRRL B-14911 and stored at 30 °C reached the crossover point, where *G*′ intersects with *G*″, at a significantly lower strain compared to the other groups (Figure 9B). These samples also displayed lower *G*′ and *G*″ modulus levels in the linear viscoelastic (LVE) region compared to the other groups (Figure 9C). These results indicate a more compliant gel structure with decreased stiffness in the presence of microbes after storage at 30 °C, while the samples stored at 4 °C did not display any loss of stiffness or viscoelasticity in the presence of microbes. Since the storage and loss modulus values were similar for bacteria-free biostickers at both temperatures, the decrease in modulus for biostickers containing bacteria at 30 °C may be due to increased metabolic activity of the microbes at the warmer temperature, perhaps via enzymatic activities. Any direct effects of temperature on the biosticker mechanical properties cannot be determined since all testing was performed at ambient temperatures.

**Figure 9:**
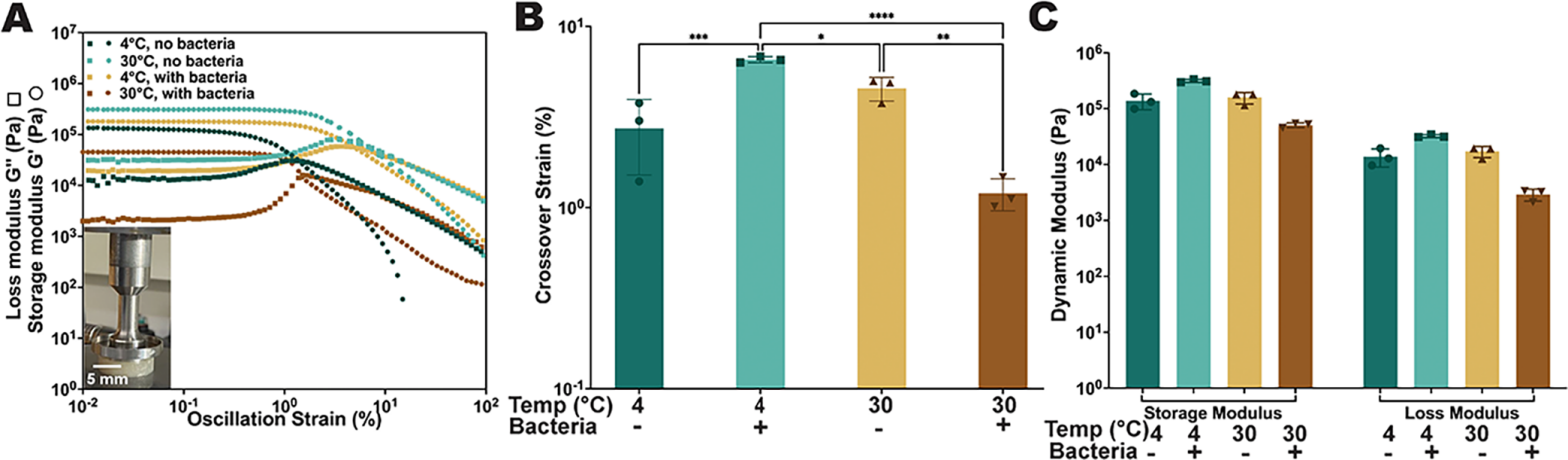
Rheological analysis of biostickers indicates viscoelastic behavior. (A) Dynamic moduli plotted against oscillation strain for biostickers, including or not including *Bacillus* sp. NRRL B-14911 and incubated at either 4 or 30 °C for 21 days. The inset image depicts the equipment setup used for testing. (B) Crossover strain values for each type of sample, indicating the critical strain where storage and loss moduli intersect (*n* = 3). (C) Storage and loss modulus values for samples in the LVE region (*n* = 3). *P ≤ 0.05, **P ≤ 0.01, ***P ≤ 0.001, ****P ≤ 0.0001 by one-way ANOVA statistical analysis.

To assess the tensile strength, stiffness, and toughness of the biostickers, monotonic tensile tests were performed to demonstrate the material’s response to uniaxial tension. Biostickers either containing *Bacillus* sp. NRRL B-14911 or not containing microbes were 3D-bioprinted into “dog-bone” structures following the ASTM D638-05 standard.^41^ The biostickers were incubated on Marine Broth-PHB agar plates for 21 days at either 4 or 30 °C prior to tensile testing (Figure S10). The stress–strain curves for each of the biosticker samples displayed an initial region in which the stress increased proportionally with strain, followed by a decrease in slope, and then fracture (Figures S10B and S11). The modulus of elasticity, which characterizes the material’s stiffness, was determined from the slope of the stress–strain curve for each material. The elastic modulus for each of the biosticker samples was around 1 MPa (Figure S10C). No significant differences in elastic modulus were seen when the biostickers were stored at 4 °C versus 30 °C, or for biostickers with or without *Bacillus* sp. NRRL B-14911. The ultimate tensile strength (UTS) of the samples was determined as the maximum tensile load that the materials could endure before failure. The biosticker samples incubated at 30 °C, both with and without microbes, demonstrated significantly higher UTS values than the biostickers incubated at 4 °C (Figure S10D). The inclusion of bacteria in the biostickers resulted in a significant decrease in UTS for biostickers incubated at 30 °C but not at 4 °C (Figure S10D), analogously to the decreased storage and loss moduli seen only at 30 °C in the rheology data, which could again perhaps be attributable to higher bacterial metabolic activity at the higher temperature.

In order to efficiently biodegrade PHB objects, biostickers would need to initially adhere to PHB materials to position their embedded PHB-degrading microbes in close proximity to the target bioplastics. To assess the adhesion strength of biostickers when bonded to PHB plates, adhesion tests were conducted. Biostickers either containing *Bacillus* sp. NRRL B-14911 or not containing microbes were 3D-bioprinted into rectangular structures and incubated on Marine Broth-agar plates for 21 days at either 4 or 30 °C prior to adhesion testing (Figure 10A). Inspired by lap shear testing protocols,^42^ PHB plates and biosticker samples were positioned in opposite grips of a universal testing machine with an overlap distance of 20 mm. The PHB plate and the biosticker were placed into direct contact through gentle pressure, and then the applied force was measured while the PHB and biostickers were vertically displaced from each other at a rate of 1 mm/s (Figures 10B and S12). The maximum load value, representing the peak force that the biostickers could endure before separating from the PHB surface, is directly proportional to the adhesion strength. The data indicated that the average maximum load values for all samples fell within the range 0.15–0.2 N (Figure 10C). The energy dissipation value, calculated from the area under the force–displacement curve, indicates the energy expended by the biosticker until interface failure. An increased energy dissipation capacity is typically associated with more durable and dependable adhesion under diverse conditions.^61^ The results showed that average energy dissipation values for all sample types ranged between 0.09 and 0.17 mJ (Figure 10D). Notably, the presence or absence of the *Bacillus* sp. NRRL B-14911 and the variations in incubation temperature did not have any significant effects on the measured adhesive properties of the biostickers (Figure 10C,D). The adhesiveness of the biostickers likely derives from a combination of its physical and chemical properties. The relatively soft and compliant biosticker material could allow it to make good physical contact with a substrate, while the carboxyl groups of the alginate polymers could provide ionic or dipole bonding interactions with a substrate.

**Figure 10:**
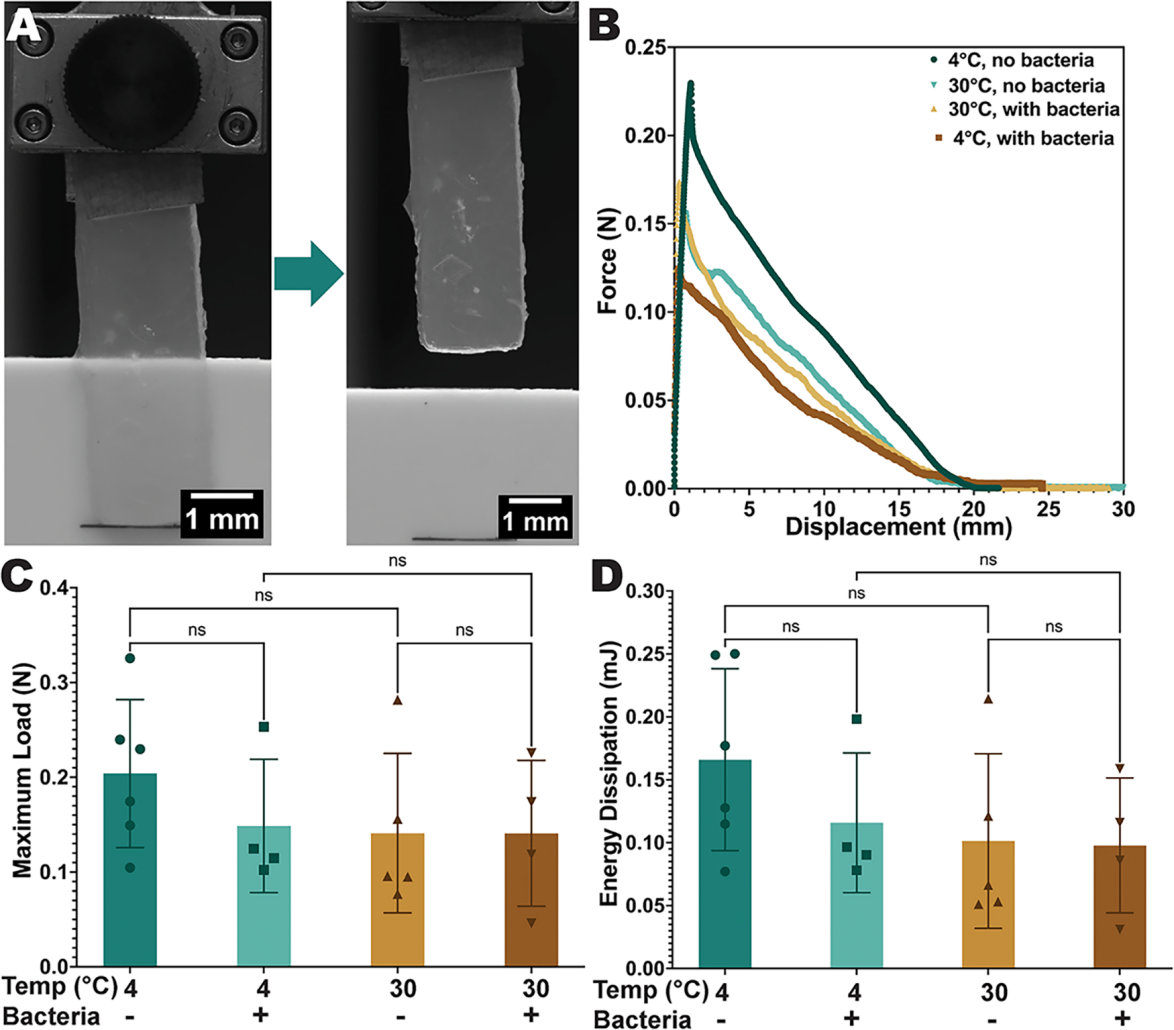
Adhesion testing of biostickers. (A) Image of a biosticker undergoing an adhesion test. (B) Force–displacement curves for biostickers including or not including *Bacillus* sp. NRRL B-14911 and incubated at either 4 or 30 °C for 21 days. (C) The maximum load for each type of sample and (D) the energy dissipation for each type of sample (*n* = 4–6). ns = not significant by one-way ANOVA statistical analysis.

## Discussion

In this study, a 3D-bioprinted “biosticker” has been developed, consisting of marine bacteria integrated within an alginate hydrogel, for accelerating and tuning the degradation of PHB bioplastics. The bioprinted marine bacteria showed viability for more than 3 weeks postprinting in salinity that mimics ocean water. Biostickers could accelerate mass loss of PHB sheets in both air and liquid environments, highlighting that the use of engineered living materials can maintain a favorable environment for the encapsulated cells under ambient conditions as well as marine-like environments. The biostickers exhibited sustained PHB depolymerization activity over several weeks, and their rate of depolymerization could be tuned by altering various 3D-bioprinting and environmental parameters. Biostickers demonstrated rapid, reversible adhesion to a variety of surfaces and retained their biodegradation activity after being transferred to a different target object. The biostickers were viscoelastic, and their elastic modulus and adhesion were not negatively affected by either temperature or microbial growth over time. These biostickers represent ELMs that can degrade plastics in marine-mimicking conditions, creating possibilities for reducing marine debris and improving the sustainability of marine-deployable equipment.

This work is a proof of concept to show the PHB degradation ability of 3D-bioprinted ELMs. The method of 3D-bioprinting of the biostickers can offer customizable shapes and dimensions,^62–64^ allowing for automated and on-demand printing of additional sizes and shapes of biostickers without the need to produce an additional mold to form the biostickers each time a different type of target object will be degraded. The biostickers are weakly and reversibly adhesive, analogously to sticky notes, allowing for transfer to different target objects when needed. Future improvements of the biosticker hydrogels could focus on characterizing and enhancing biosticker adhesion following submergence in seawater or on embedding 3D-bioprinted PHB-degrading hydrogels within PHB objects to enhance their durability in ocean waters. The microbe utilized in these biostickers, *Bacillus* sp. NRRL B-14911, was isolated from the Gulf of Mexico;^35^ deployment of the biostickers in different marine ecosystems may necessitate the use of different native microbes so as not to disrupt the local microbiomes. The highest rates of PHB degradation were observed at 30 °C, representing temperatures found near the surface of tropical ocean waters.^46^ Since nontropical and subsurface regions of the ocean are cooler than 30 °C, future work will entail identifying or engineering bacterial strains capable of high rates of PHB biodegradation at lower temperatures, possibly by leveraging psychrotolerant or deep-sea microbes. The coldest regions of the ocean range from −2 to 3 °C,^65^ which may pose a particular challenge since PHB has a glass transition temperature of around 4 °C.^66^ Below this temperature, the PHB polymer chain mobility and water penetration will be decreased, likely further slowing biodegradation rates.

## Conclusions

Overall, this work demonstrates a first step toward developing scalable and tunable strategies to mitigate ocean debris while integrating eco-friendly materials into the rapidly expanding blue economy. By addressing the inherent challenges to plastic degradation posed by marine environments, 3D-bioprinted biostickers could be further developed to help transform the fate of bioplastic objects, enabling their responsible disposal. The potential to extend these methods to other biodegradable plastic polymers as well as to apply or engineer additional microbial strains with enhanced bioplastic degradation activity in a range of ocean-relevant conditions, will be interesting for future research. Ultimately, these advancements are hoped to promote autonomous marine sensing and Internet of Things (IoT) deployments in ways that reduce long-term ecological impact.

## Supplementary Material





## Notes

Logistic fitting methods for viability and degradation rates; clear zone assay for PHB biodegradation; growth and viability of *Bacillus* sp. NRRL B-14911 under different environmental and bioprinting conditions; preparation of samples for SEM analysis of biodegradation of PHB sheets; pH of biostickers during biodegradation of PHB sheets; progression of PHB degradation by biostickers with varied geometries; PHB degradation testing by biostickers vs free cells; clear zone and CFU assays for biostickers with altered parameters; rheometry, tensile testing, and adhesion data for biostickers (PDF)
Biostickers are transferred to different plates without breaking, adhered directly to the tweezers and to the new Marine Broth-PHB agar surface (Video S1) (MP4)
