# Supplementary material for: 3D-Bioprinted Marine Bacteria for the Degradation of Polyhydroxybutyrate Bioplastics
Source: ACS Appl Polym Mater. 2026 Apr 25;8(9):6086–100. doi: 10.1021/acsapm.5c03370 (PMC13162202; doi:10.1021/acsapm.5c03370)
Supplement: Supplementary file 1 [file ap5c03370_si_001.pdf]

Supporting Information for Publication

3D-bioprinted marine bacteria for the degradation of polyhydroxybutyrate bioplastics

*Luying He[1], Hongyi Cai[2], Ram S. Gona[3], Tiana C. Rohe[4], Manasi Subhash Gangan[1], Timothy Lai[1], Diana R. Sullivan[5], Meredith N. Silberstein[6], and Anne S. Meyer[1,3]**

[1] Department of Biology, University of Rochester, Rochester, New York, 14627, United States of America

[2] Materials Science and Engineering, Cornell University, Ithaca, New York, 14853, United States of America

[3] Program of Materials Science, University of Rochester, Rochester, New York, 14627, United States of America

[4] Department of Biochemistry and Biophysics, University of Rochester, Rochester, New York, 14627, United States of America

[5] Department of Chemical Engineering, University of Rochester, Rochester, New York, 14627, United States of America

[6] Sibley School of Mechanical and Aerospace Engineering, Cornell University, Ithaca, New York, 14853, United States of America

Engineered Living Materials Institute, Cornell University, Ithaca, New York, 14853, United States of America

* E-mail: anne.meyer@rochester.edu

Supporting Methods

Logistic fitting of the viability and PHB degradation rates of 3D-bioprinted Bacillus sp. NRRL B-14911.

The survival of 3D-bioprinted *Bacillus* sp. NRRL B-14911 was analyzed quantitatively. CFU data over time was fitted to a logistic bacterial growth equation[1] (Equation 1) using the cftool

app in MATLAB R2022b (MathWorks), to obtain estimates of growth rate (r), maximum growth rate (k), and initial growth rate (N).

$$f(x) = (N * k)/(N + ((k - N) * \exp(-r * x))) \qquad (1)$$

Parameter estimates with 95% confidence intervals are: N = 0.0002769 (0.0001325, 0.0004213), k = 0.05186 (0.04125, 0.06248), and r = 0.2659 (0.2283, 0.3034). The sum of squared estimate of errors (SSE) is $2.504e^{-6}$, R^2 is 0.9981, and root mean square error is 0.0005595.

The PHB degradation rate of bio-stickers containing *Bacillus* sp. NRRL B-14911 was analyzed quantitatively. PHB degradation data by bio-stickers over time with varying concentrations of PHB was fitted to a logistic growth equation (Equation 1) to obtain estimates of degradation rate (r), maximum degradation rate (k), and initial degradation rate (N). Parameter estimates with 95% confidence intervals are: N = 0.2002 (-1.607, 2.007), k = 6.685 (-1.092, 14.46), and r = 1.31 (-2.635, 5.255). SSE is 8.868, R^2 is 0.6299, and root mean square error is 1.719.

Supporting References

(1) Hajmeer, M.; Basheer, I. Comparison of logistic regression and neural network-based classifiers for bacterial growth. *Food Microbiology* **2003**, *20* (1), 43-55. DOI: 10.1016/S0740-0020(02)00104-1.

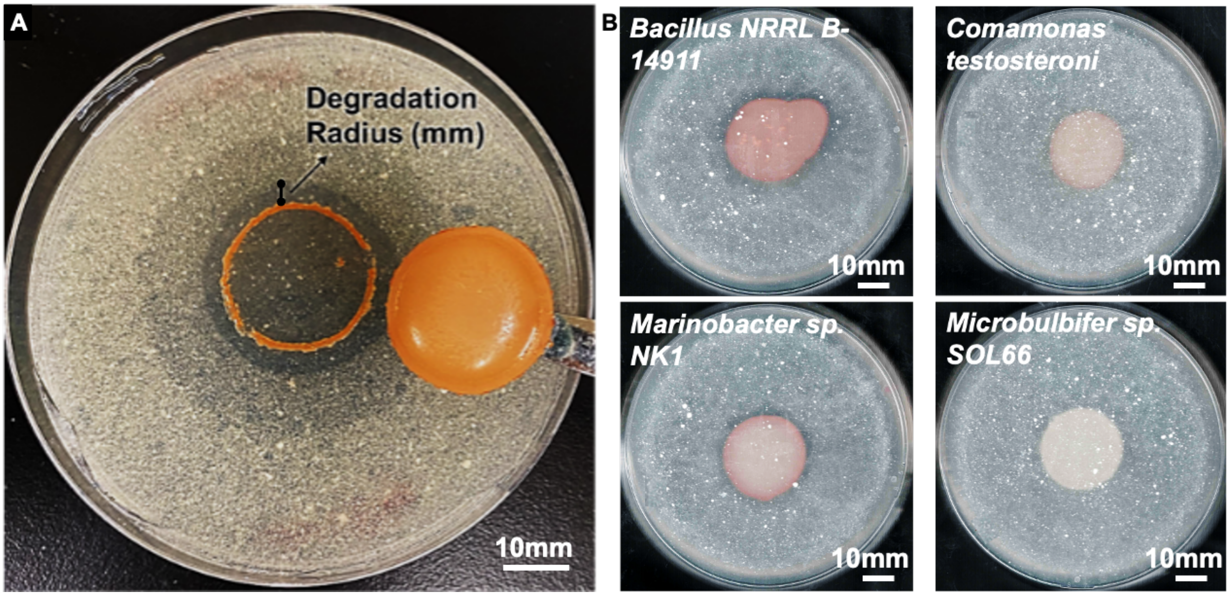

Figure S1. Clear-zone assay for PHB biodegradation. (A) Degradation of PHB in a Marine Broth-agar plate by a bio-sticker (orange) containing *Bacillus* sp. NRRL B-14911. PHB degradation can be visualized via the disappearance of opaque PHB powder and the appearance of clear zones within the agar. (B) Representative clear-zone assay samples for liquid cultures of *Bacillus* sp. NRRL B-14911, *Comamonas testosteroni*, *Marinobacter* sp. NK-1, or *Microbulbifer* sp. SOL66, pipetted onto Marine Broth-agar plates supplemented with 0.3 M $CaCl_2$ and 0.2 % (w/v) PHB powder; plates were incubated at 30 °C for 7 days (n = 4).

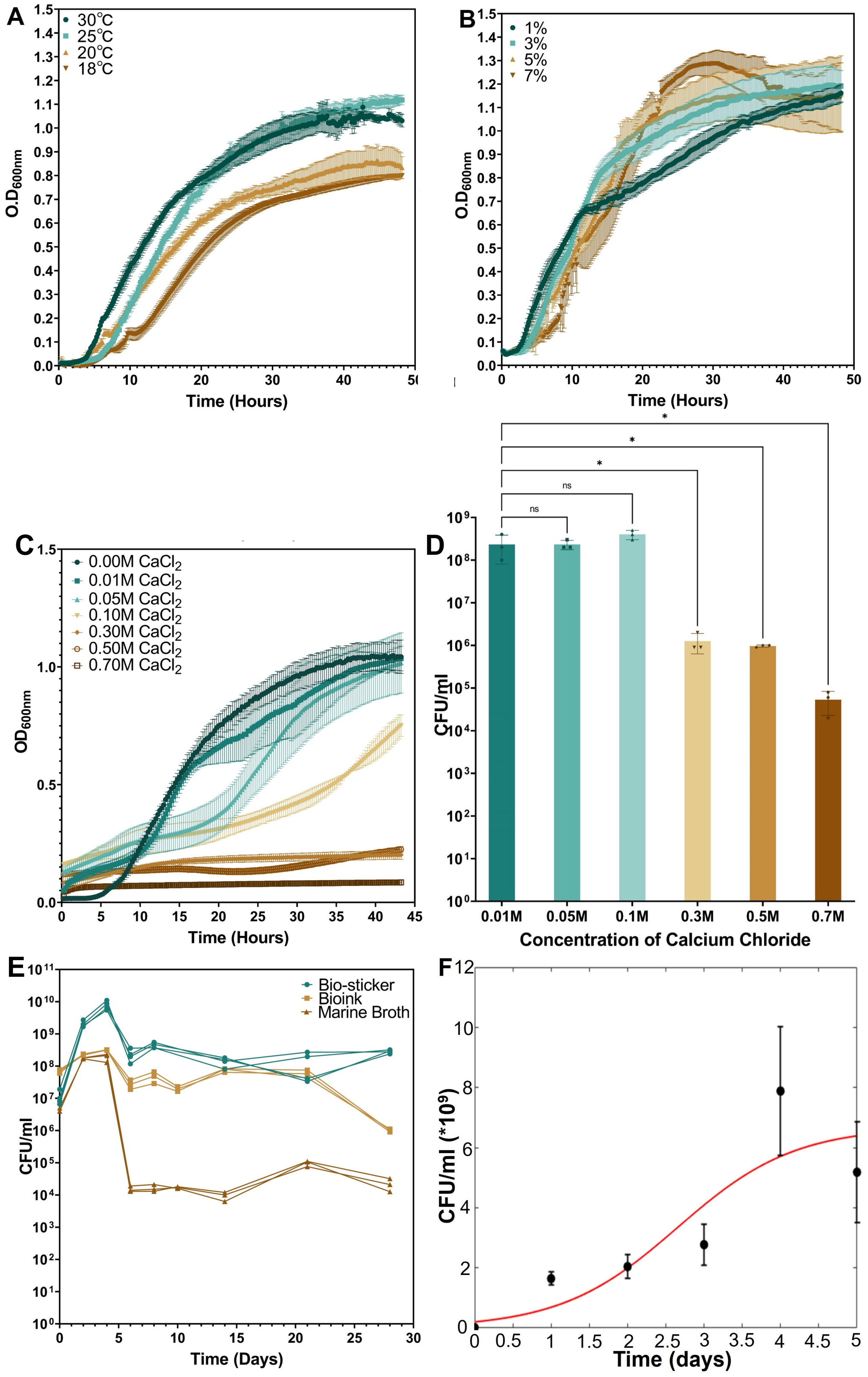

Figure S2. Growth and viability of *Bacillus* sp. NRRL B-14911 under different environmental and bio-printing conditions. (A) Growth curves of *Bacillus* sp. NRRL B-14911

cultured in Marine Broth at 30 °C (dark green), 25 °C (light green), 20 °C (light brown), and 18 °C (dark brown). (n=3) (B) Growth curves of *Bacillus* sp. NRRL B-14911 cultured in LB media supplemented with sodium chloride at 1% (dark green), 3% (light green), 5% (light brown), or 7% (dark brown). (n=3) (C) Growth curves of *Bacillus* sp. NRRL B-14911 cultured in Marine Broth supplemented with $CaCl_2$ at concentrations ranging from 0.00 M (darkest green) to 0.70 M (darkest brown). (n=3) (D) CFUs of *Bacillus* sp. NRRL B-14911 in bio-stickers 3D-bioprinted onto Marine Broth-agar plates containing $CaCl_2$ at concentrations ranging from 0.01 M (darkest green) to 0.7 M (darkest brown). (n=3) * $P \leq 0.05$, ns = not significant by one-way ANOVA statistical analysis. (E) CFU/mL of *Bacillus* sp. NRRL B-14911 following incubation in liquid Marine Broth (dark brown), liquid bio-ink (light brown), and polymerized bio-ink 3D-bioprinted into bio-stickers (dark green) over 28 days. (n=3). (F) CFU/mL of *Bacillus* sp. NRRL B-14911 over 5 days of growth within a 3D-biopriinted bio-sticker (black circles) was fit to a logistic equation. (n=3)

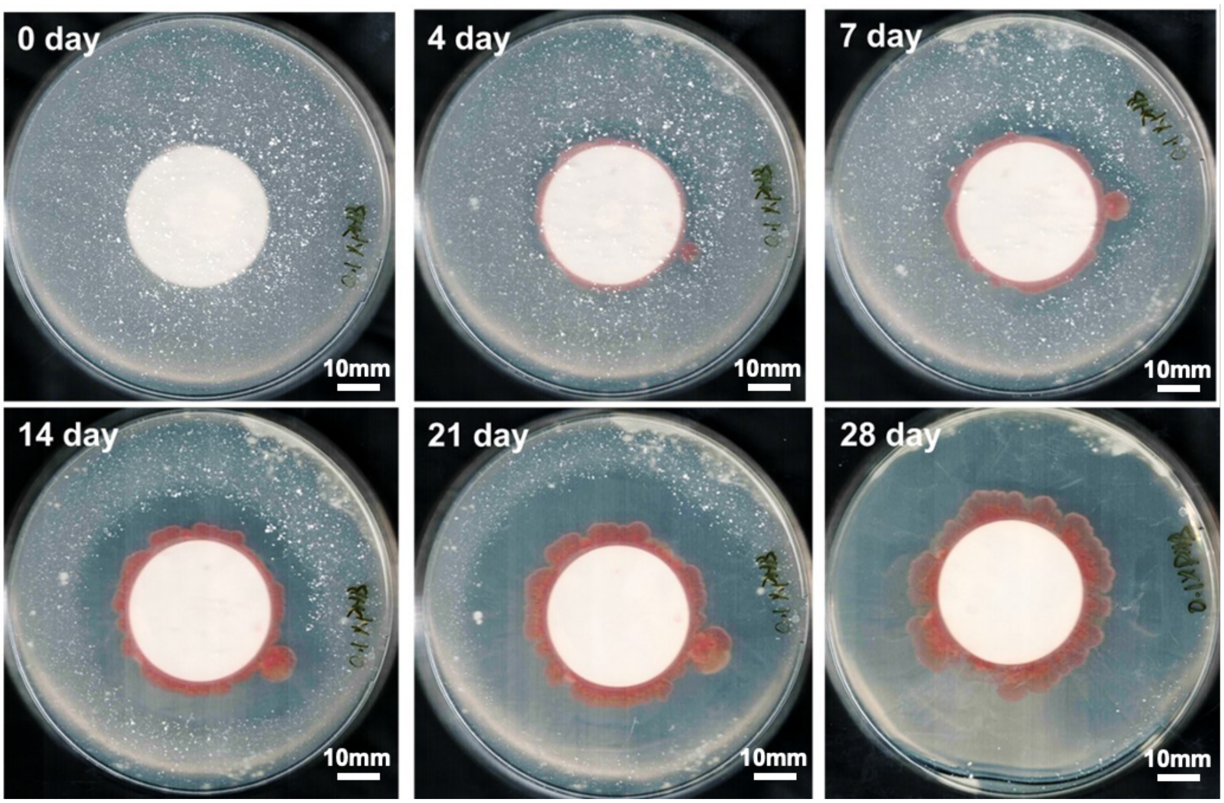

Figure S3. Preparation of samples for SEM analysis of bio-degradation of PHB sheets. Solid PHB discs (1-mm thickness, 44.5-mm diameter) were placed onto Marine Broth agar plates containing 0.1% PHB. Bio-stickers were applied overtop of the PHB discs, and samples were incubated at 30 °C for 28 days. At the end of the incubation period, the PHB discs were carefully removed, and residual bacteria and excess hydrogel were meticulously cleansed from their surfaces. PHB was added to these agar plates at a lower concentration than typical to allow for monitoring the appearance of clear zones which would indicate whether the samples were contaminated with unrelated bacteria strains, since contaminants would likely not be able to depolymerize PHB.

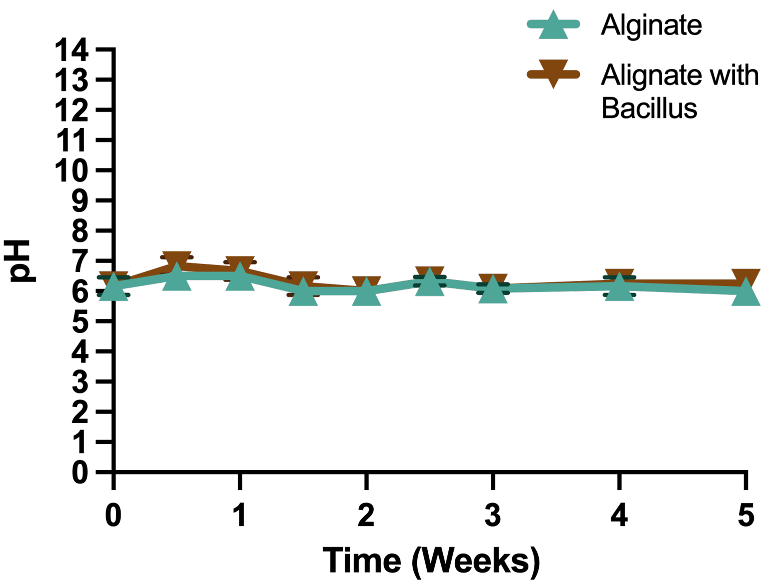

Figure S4. pH of bio-stickers during bio-degradation of PHB sheets. pH measurements of bio-stickers with or without *Bacillus* sp. NRRL B-14911 that were applied onto the surface of solid PHB sheets and incubated at 30 °C for 5 weeks.

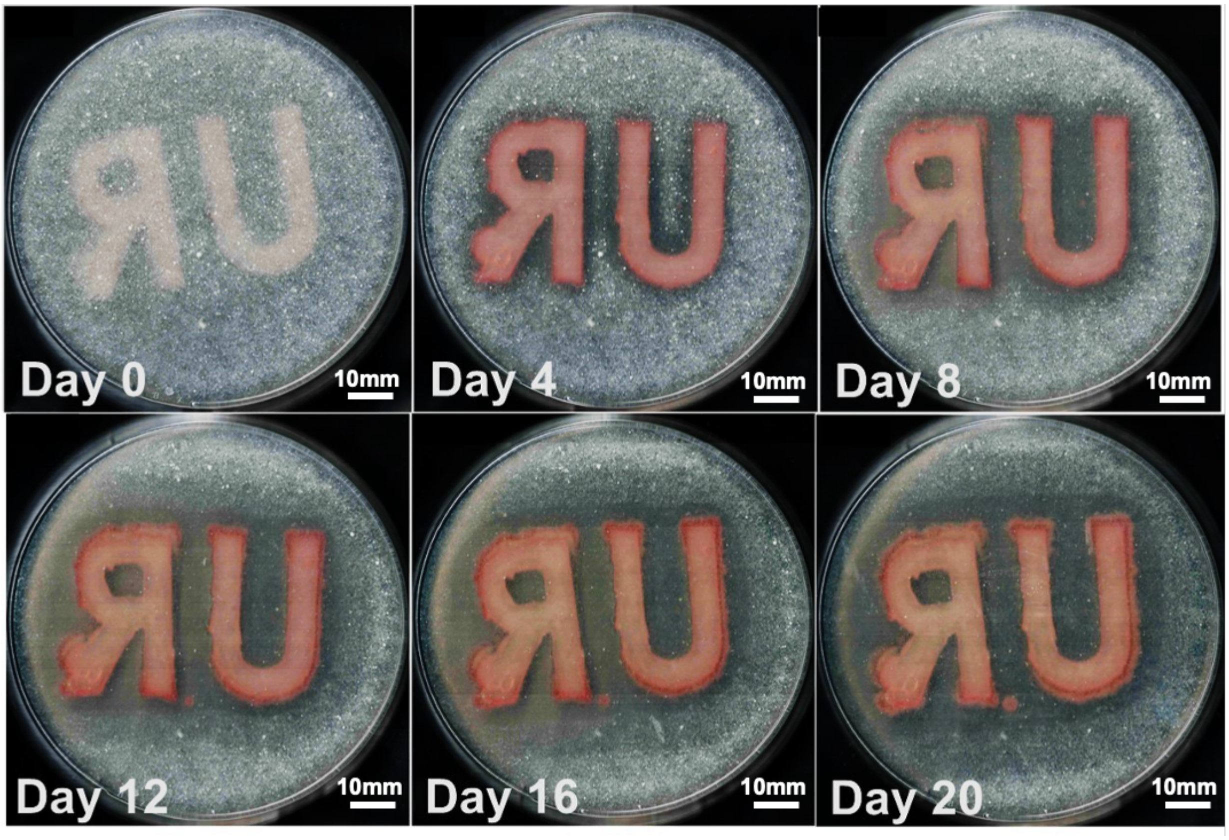

Figure S5. Progression of PHB degradation by bio-stickers with varied geometries. Bio-stickers were 3D-printed into "U" and "R" shapes and placed onto Marine Broth-PHB agar plates. Samples were incubated at 30 °C for 28 days. During this time period, the PHB was first cleared from the agar underneath the bio-stickers, after which the clear zones continually expanded in radius.

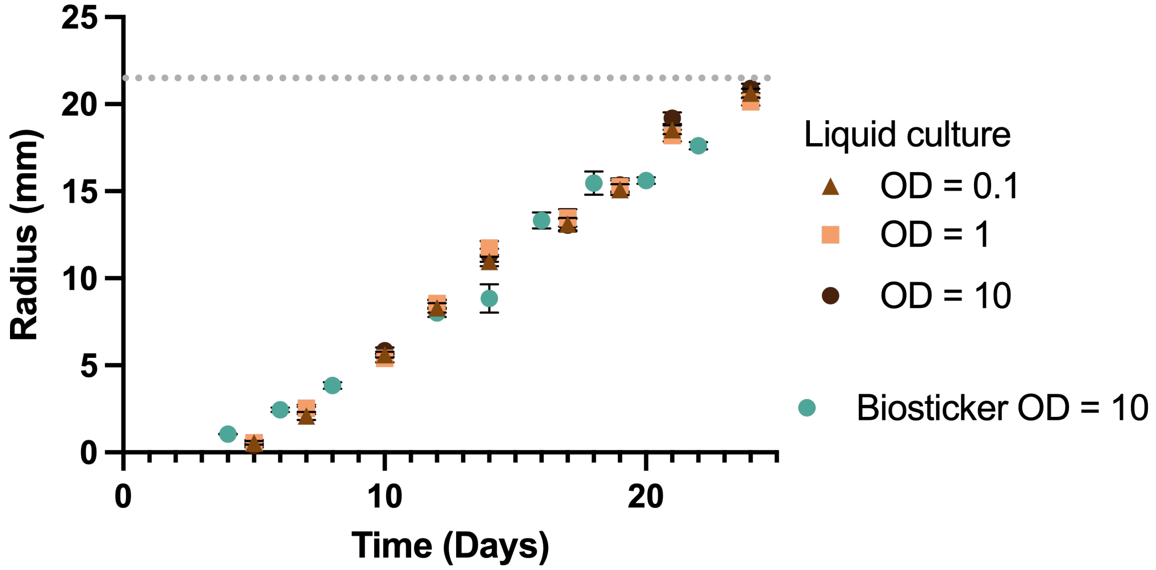

Figure S6. PHB degradation by bio-stickers vs. free bacteria cells. Clear zone radius over time for *Bacillus* sp. NRRL B-14911 in bio-stickers at $O.D._{600} = 10$ or applied from liquid culture at $O.D._{600} = 0.1$, 1, or 10. Liquid cultures were applied at a volume of 0.53 mL, which is equivalent to the volume of bio-ink in a bio-sticker. The horizontal dotted line indicates the limit of measurement for clear zones, due to plate boundaries.

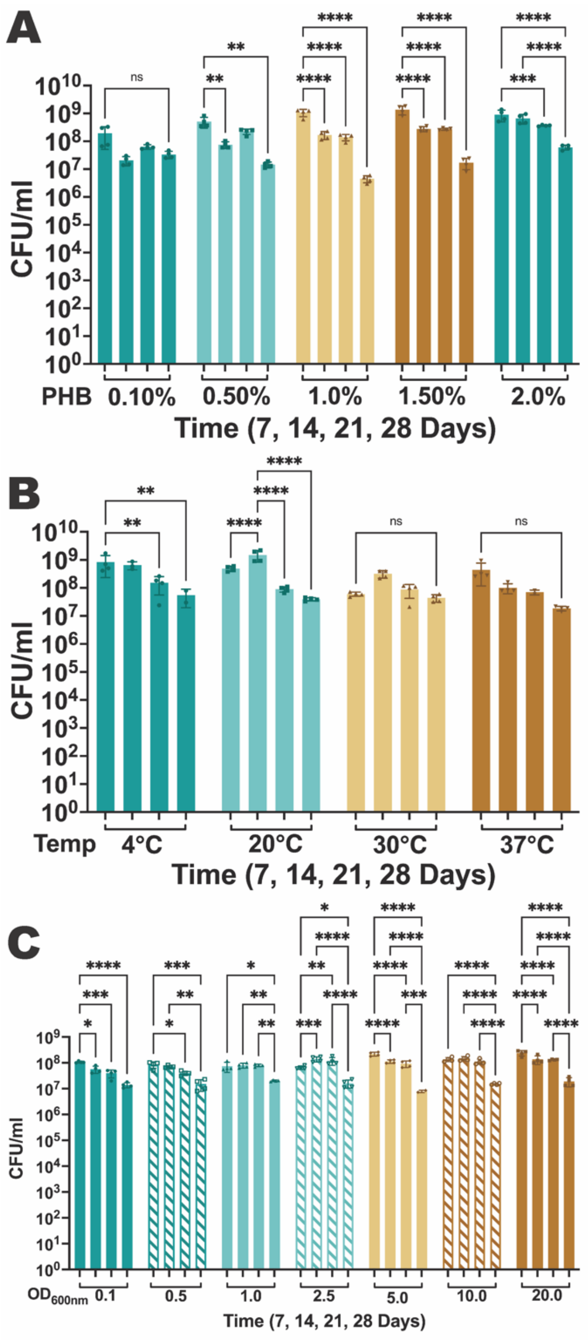

Figure S7. CFU values for bio-stickers with altered parameters. CFU assays for bio-stickers at an O.D.$_{600}$ of 10 incubated at 30 °C on Marine Broth-PHB agar plates containing 0.5% PHB powder, except where altered parameters are noted. (A) CFU/mL over 28 days for bio-stickers bio-printed onto plates containing varying PHB concentrations. (n=4) (B) CFU/mL over 28 days for bio-stickers incubated at varying temperatures. (n=4) (C) CFU/mL over 28 days for bio-stickers printed using bio-ink with varying initial O.D.$_{600}$. (n=4)

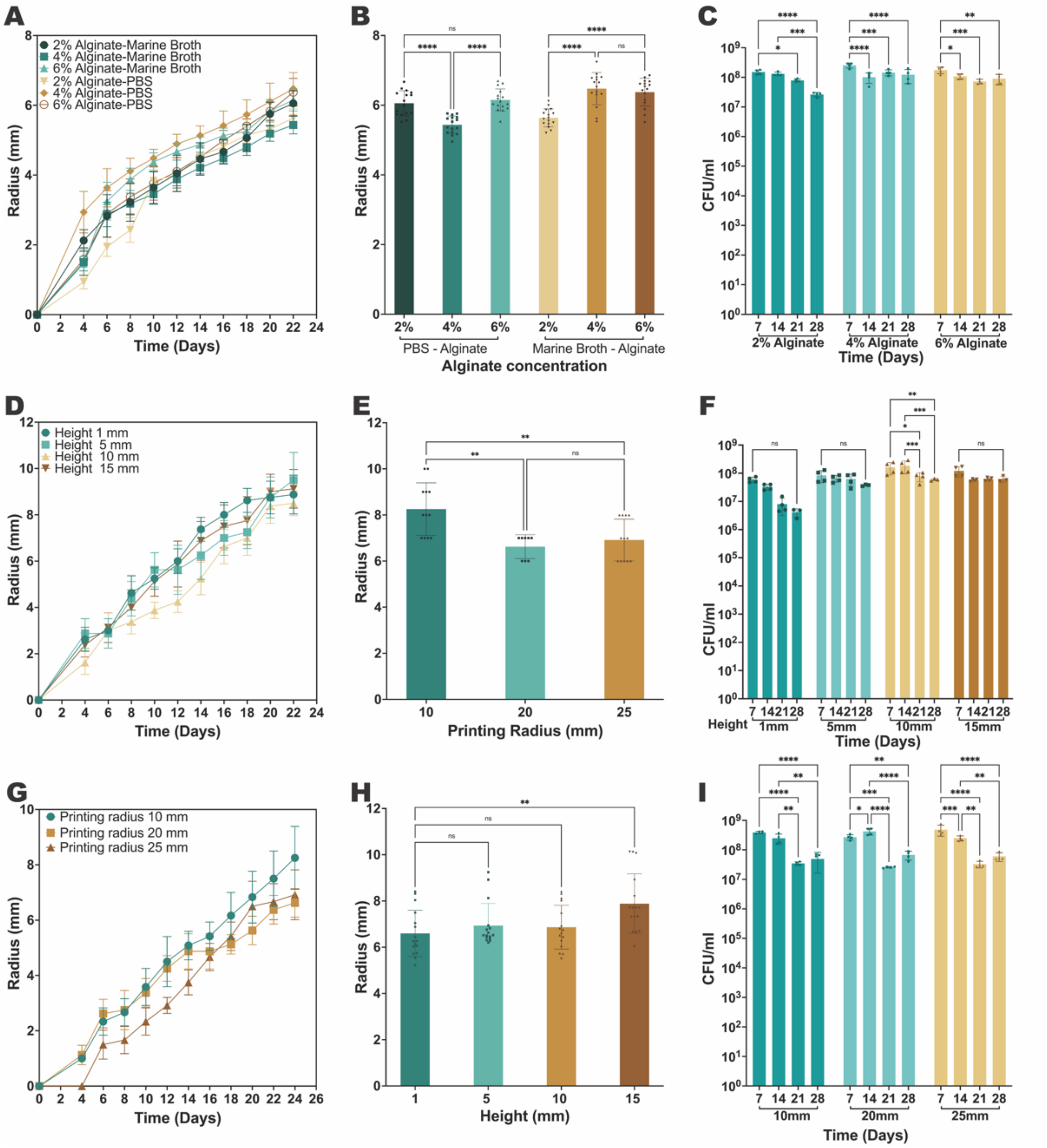

Figure S8. PHB degradation is modestly affected by tuning bio-sticker geometry and hydrogel density. Clear zone and CFU assays for bio-stickers with diameter of 10 mm and height of 2 mm, incubated on Marine Broth-PHB agar plates, except where altered parameters are noted. (A-C) Clear zone radius over time (A) and on day 22 (B) and CFU/mL over time (C) for bio-stickers containing varying alginate concentrations. CFU/mL data in panel C is for alginate-Marine Broth bio-stickers. (D-F) Clear zone radius over time (D) and on day 22 (E) and CFU/mL over time (F) for bio-stickers 3D-bioprinted with varying heights. (G-H) Clear zone radius over time (G) and on day 24 (H) and CFU/mL over time (I) for bio-stickers 3D-bioprinted with varying initial radii. Vertical dashed red lines in panels A, D, and G indicate time points that

were analyzed in panels B, E, and H for statistical differences between conditions. ** P≤0.01, **** P≤0.0001, ns = not significant by one-way ANOVA statistical analysis

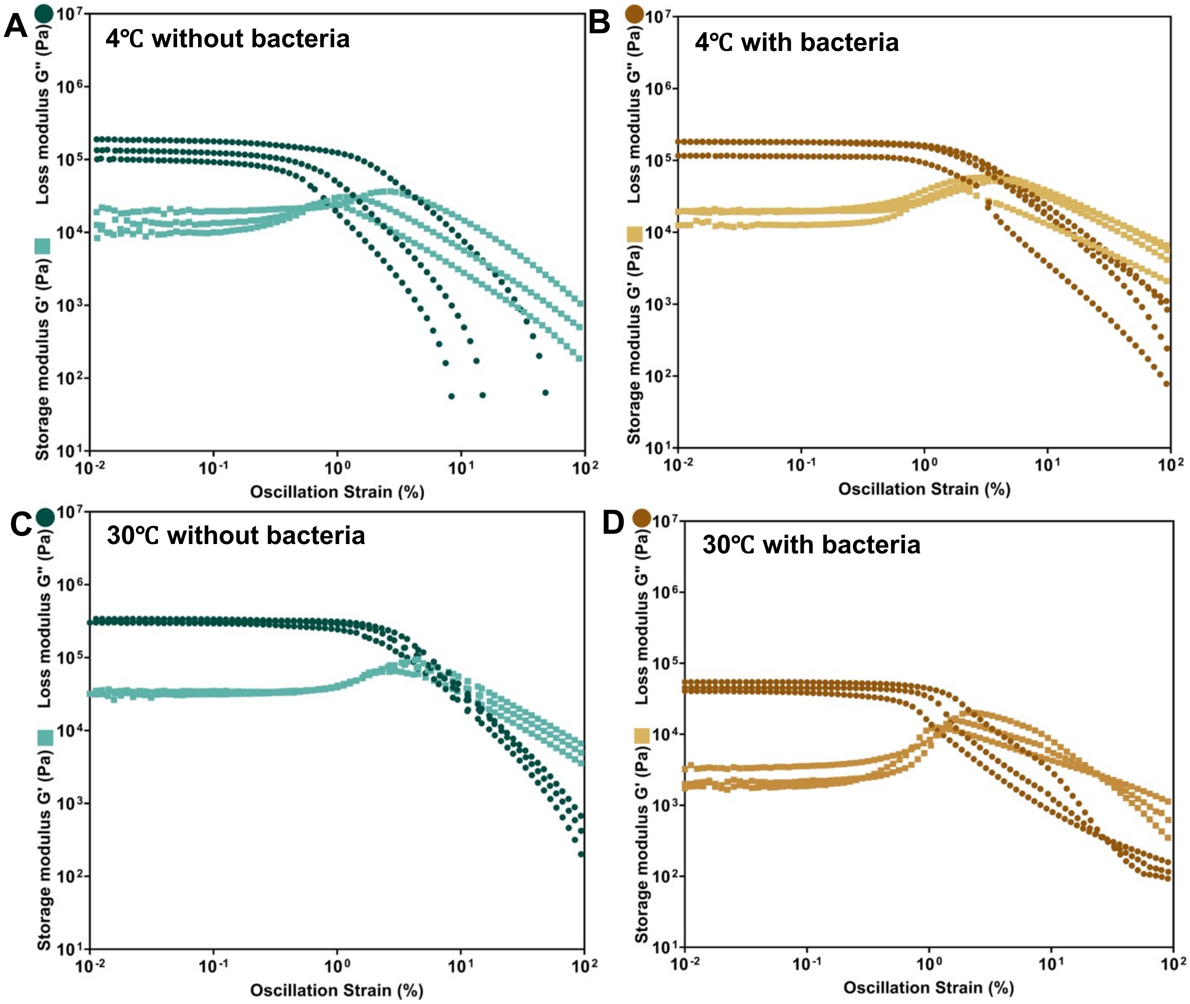

Figure S9. Rheometry data for bio-stickers. Rheometry testing data showing the relationship between oscillation strain and both storage modulus (squares) and loss modulus (circles) for (A-B) bio-stickers incubated at 4 °C and (A) not containing or (B) containing *Bacillus* sp. NRRL B-14911, and (C-D) bio-stickers incubated at 30 °C and (C) not containing or (D) containing *Bacillus* sp. NRRL B-14911.

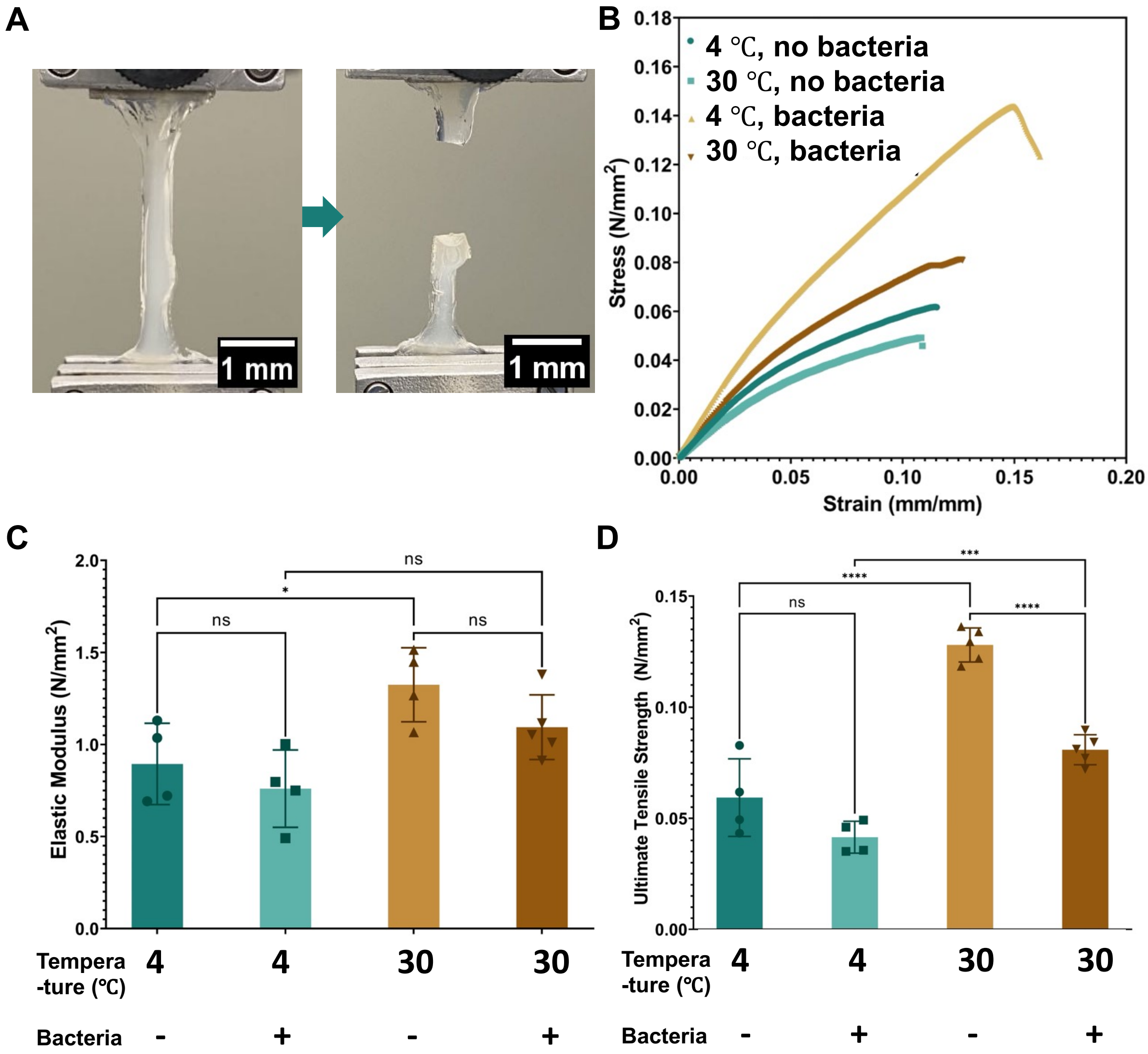

Figure S10. Tensile testing of bio-stickers. (A) Image of a 3D-printed bio-sticker undergoing a tension loading test. (B) Strain-stress curves for 3D-printed bio-stickers including or not including *Bacillus* sp. NRRL B-14911 and incubated at either 4 °C or 30 °C for 21 days. (C) The elastic modulus for each type of sample and (D) the ultimate tensile strength for each type of sample (n=4-5). * $P \leq 0.05$, *** $P \leq 0.001$, **** $P \leq 0.0001$, ns = not significant by one-way ANOVA statistical analysis

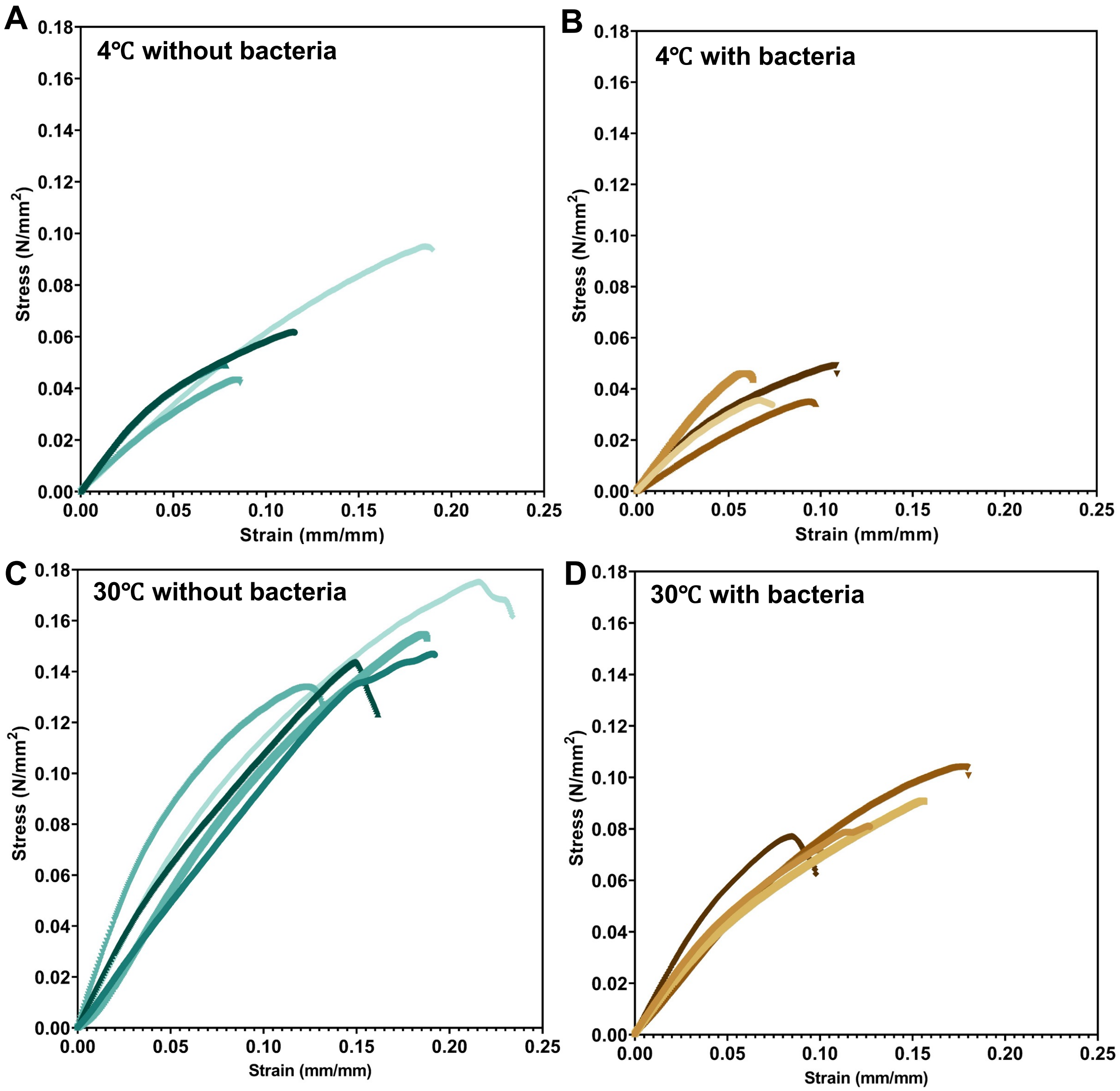

Figure S11. Tensile testing stress-strain curves for bio-stickers. (A-B) Tensile testing stress-strain curves for bio-stickers incubated at 4 °C and (A) not containing or (B) containing *Bacillus* sp. NRRL B-14911. (C-D) Tensile testing stress-strain curves for bio-stickers incubated at 30 °C and (C) not containing or (D) containing *Bacillus* sp. NRRL B-14911.

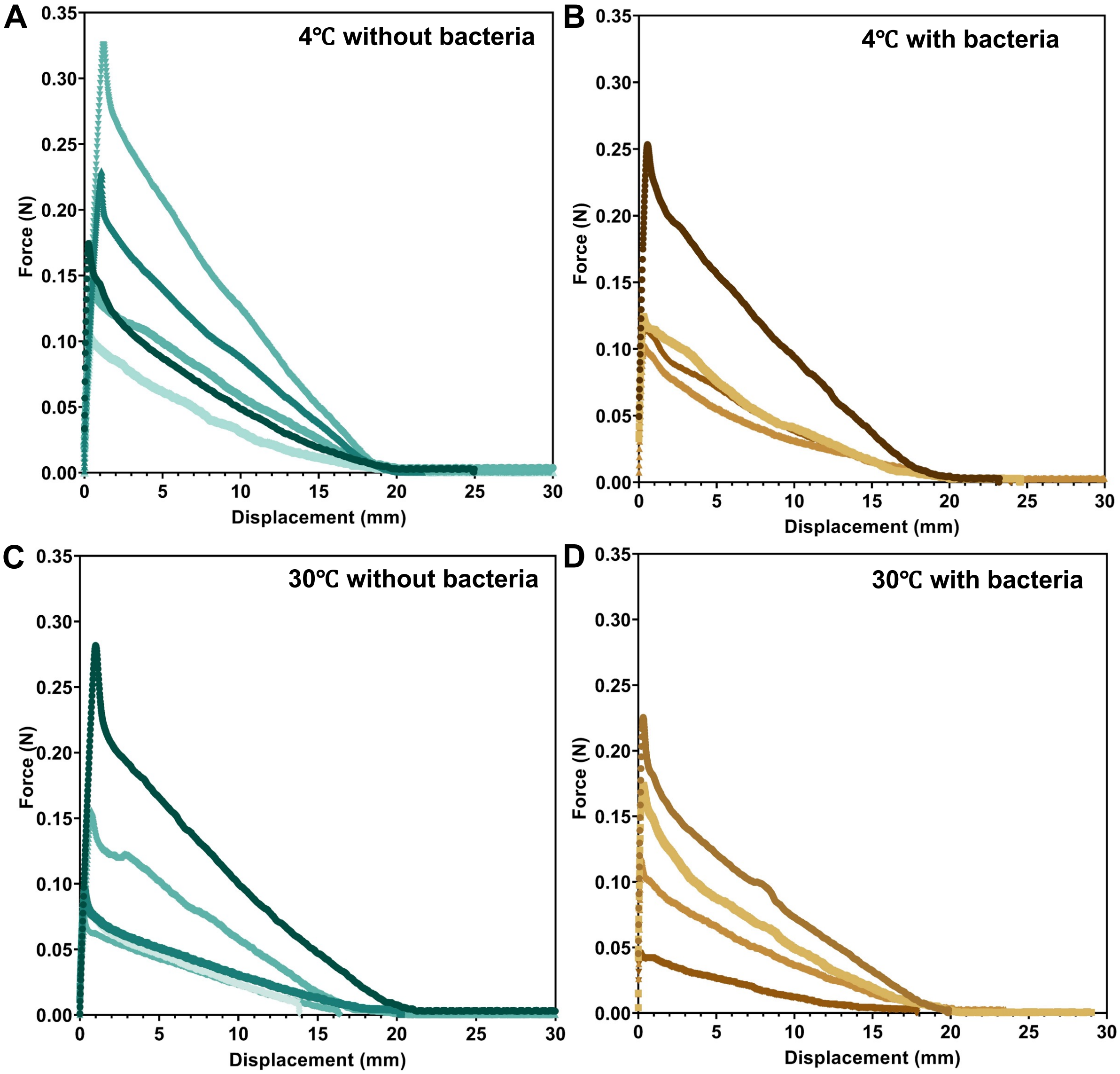

Figure S12. Adhesion testing force-displacement curves for bio-stickers. (A-B) Adhesion testing force-displacement curves for bio-stickers incubated at 4 °C and (A) not containing or (B) containing *Bacillus* sp. NRRL B-14911. (C-D) Adhesion testing force-displacement curves for bio-stickers incubated at 30 °C and (C) not containing or (D) containing *Bacillus* sp. NRRL B-14911.
